# A multidimensional systems biology analysis of cellular senescence in aging and disease

**DOI:** 10.1186/s13059-020-01990-9

**Published:** 2020-04-07

**Authors:** Roberto A. Avelar, Javier Gómez Ortega, Robi Tacutu, Eleanor J. Tyler, Dominic Bennett, Paolo Binetti, Arie Budovsky, Kasit Chatsirisupachai, Emily Johnson, Alex Murray, Samuel Shields, Daniela Tejada-Martinez, Daniel Thornton, Vadim E. Fraifeld, Cleo L. Bishop, João Pedro de Magalhães

**Affiliations:** 1 Integrative Genomics of Ageing Group, Institute of Ageing and Chronic Disease, University of Liverpool, Liverpool, L7 8TX UK; 2 School of Biological Sciences, Monash University, Melbourne, VIC 3800 Australia; 3 Computational Biology of Aging Group, Institute of Biochemistry, Romanian Academy, 060031 Bucharest, Romania; 4 Chronos Biosystems SRL, 060117 Bucharest, Romania; 5 Centre for Cell Biology and Cutaneous Research, Blizard Institute, Barts and The London School of Medicine and Dentistry, Queen Mary University of London, London, E1 2AT UK; 6 Research and Development Authority, Barzilai Medical Center, Ashkelon, Israel; 7 Doctorado en Ciencias mención Ecología y Evolución, Instituto de Ciencias Ambientales y Evolutivas, Facultad de Ciencias, Universidad Austral de Chile, Independencia 631, Valdivia, Chile; 8 The Shraga Segal Department of Microbiology, Immunology and Genetics, Faculty of Health Sciences, Center for Multidisciplinary Research on Aging, Ben-Gurion University of the Negev, 8410501 Beer Sheva, Israel

**Keywords:** Biogerontology, Cancer, Genetics, Longevity, Transcriptome

## Abstract

**Background:**

Cellular senescence, a permanent state of replicative arrest in otherwise proliferating cells, is a hallmark of aging and has been linked to aging-related diseases. Many genes play a role in cellular senescence, yet a comprehensive understanding of its pathways is still lacking.

**Results:**

We develop CellAge (http://genomics.senescence.info/cells), a manually curated database of 279 human genes driving cellular senescence, and perform various integrative analyses. Genes inducing cellular senescence tend to be overexpressed with age in human tissues and are significantly overrepresented in anti-longevity and tumor-suppressor genes, while genes inhibiting cellular senescence overlap with pro-longevity and oncogenes. Furthermore, cellular senescence genes are strongly conserved in mammals but not in invertebrates. We also build cellular senescence protein-protein interaction and co-expression networks. Clusters in the networks are enriched for cell cycle and immunological processes. Network topological parameters also reveal novel potential cellular senescence regulators. Using siRNAs, we observe that all 26 candidates tested induce at least one marker of senescence with 13 genes (*C9orf40*, *CDC25A*, *CDCA4*, *CKAP2*, *GTF3C4*, *HAUS4*, *IMMT*, *MCM7*, *MTHFD2*, *MYBL2*, *NEK2*, *NIPA2*, and *TCEB3*) decreasing cell number, activating p16/p21, and undergoing morphological changes that resemble cellular senescence.

**Conclusions:**

Overall, our work provides a benchmark resource for researchers to study cellular senescence, and our systems biology analyses reveal new insights and gene regulators of cellular senescence.

**Electronic supplementary material:**

**Supplementary information** accompanies this paper at 10.1186/s13059-020-01990-9.

## Background

In the 1960s, Leonard Hayflick and Paul Moorhead demonstrated that human fibroblasts reached a stable proliferative growth arrest between their fortieth and sixtieth divisions [1]. Such cells would enter an altered state of “replicative senescence,” subsisting in a non-proliferating, metabolically active phase with a distinct vacuolated morphology [2]. This intrinsic form of senescence is driven by gradual replicative telomere erosion, eventually exposing an uncapped free double-stranded chromosome end and triggering a permanent DNA damage response [3, 4]. Additionally, acute premature senescence can occur as an antagonistic consequence of genomic, epigenomic, or proteomic damage, driven by oncogenic factors, oxidative stress, or radiation [5]. Initially considered an evolutionary response to reduce mutation accrual and subsequent tumorigenesis, the pleiotropic nature of senescence has also been positively implicated in processes including embryogenesis [6, 7], wound healing [8], and immune clearance [9, 10]. By contrast, the gradual accumulation and chronic persistence of senescent cells with time promotes deleterious effects that are considered to accelerate deterioration and hyperplasia in aging [11]. Senescent cells secrete a cocktail of inflammatory and stromal regulators—denoted as the senescence-associated secretory phenotype, or SASP—which adversely impact neighboring cells, the surrounding extracellular matrix, and other structural components, resulting in chronic inflammation, the induction of senescence in healthy cells, and vulnerable tissue [12, 13]. Mice expressing transgenic INK-ATTAC, which induces apoptosis of p16-positive senescent cells, also have increased lifespan and improved healthspan [14]. It is, therefore, no surprise that in recent years gerontology has heavily focused on the prevention or removal of senescent cells as a means to slow or stop aging and related pathologies [15–17].

Research has sought to ascertain the genetic program and prodrome underlying the development and phenotype of senescent cells [18]. Expedited by recent advances in genomic and transcriptomic sequencing, alongside high-throughput genetic screens, a wealth of publicly available data now exists which has furthered the understanding of senescence regulation [19, 20]. Unfortunately, despite our increasing knowledge of cellular senescence (CS), determining whether a cell has senesced is not clear-cut. Common senescence markers used to identify CS in vitro and in vivo include senescence-associated β-galactosidase (SA-β-gal) and p16^INK4A^ (p16) [21–23]. However, β-galactosidase activity has been detected in other cell types such as macrophages, osteoclasts, and cells undergoing autophagy [24–26]. Furthermore, some forms of senescence are not associated with p16 expression, while p16 has been detected in non-senescent cells [3, 27]. As such, there are now over 200 genes implicated in CS in humans alone. Therefore, it is necessary to conglomerate this data into a purposefully designed database.

Gene databases are highly useful for genomic computational analyses, as exemplified by the Human Ageing Genomic Resources (HAGR) [28]. HAGR provides databases related to the study of aging, including the GenAge database of aging-related genes, which contains genes related to longevity and aging in model organisms and humans, and DrugAge, which includes a compilation of drugs, compounds, and supplements that extend lifespan in model organisms. CellAge builds on these HAGR facilities to provide a means of studying CS in the context of aging or as a standalone resource; the expectation is that CellAge will now provide the basis for processing the discrete complexities of cellular senescence on a systematic scale.

Our recent understanding of biological networks has led to new fields, like network medicine [29]. Biological networks can be built using protein interaction and gene co-expression data. A previous paper used protein-protein interactions to build genetic networks identifying potential longevity genes along with links between genes and aging-related diseases [30]. Here, we present the network of proteins and genes co-expressed with the CellAge senescence genes. Assaying the networks, we find links between senescence and immune system functions and find genes highly connected to CellAge genes under the assumption that a guilt-by-association approach will reveal genes with similar functions [31].

In this study, we look at the broad context of CS genes—their association with aging and aging-related diseases, functional enrichment, evolutionary conservation, and topological parameters within biological networks—to further our understanding of the impact of CS in aging and diseases. Using our networks, we generate a list of potential novel CS regulators and experimentally validate 26 genes using siRNAs, identifying 13 new senescence inhibitors.

## Results

### The CellAge database

**Fig. 1:**
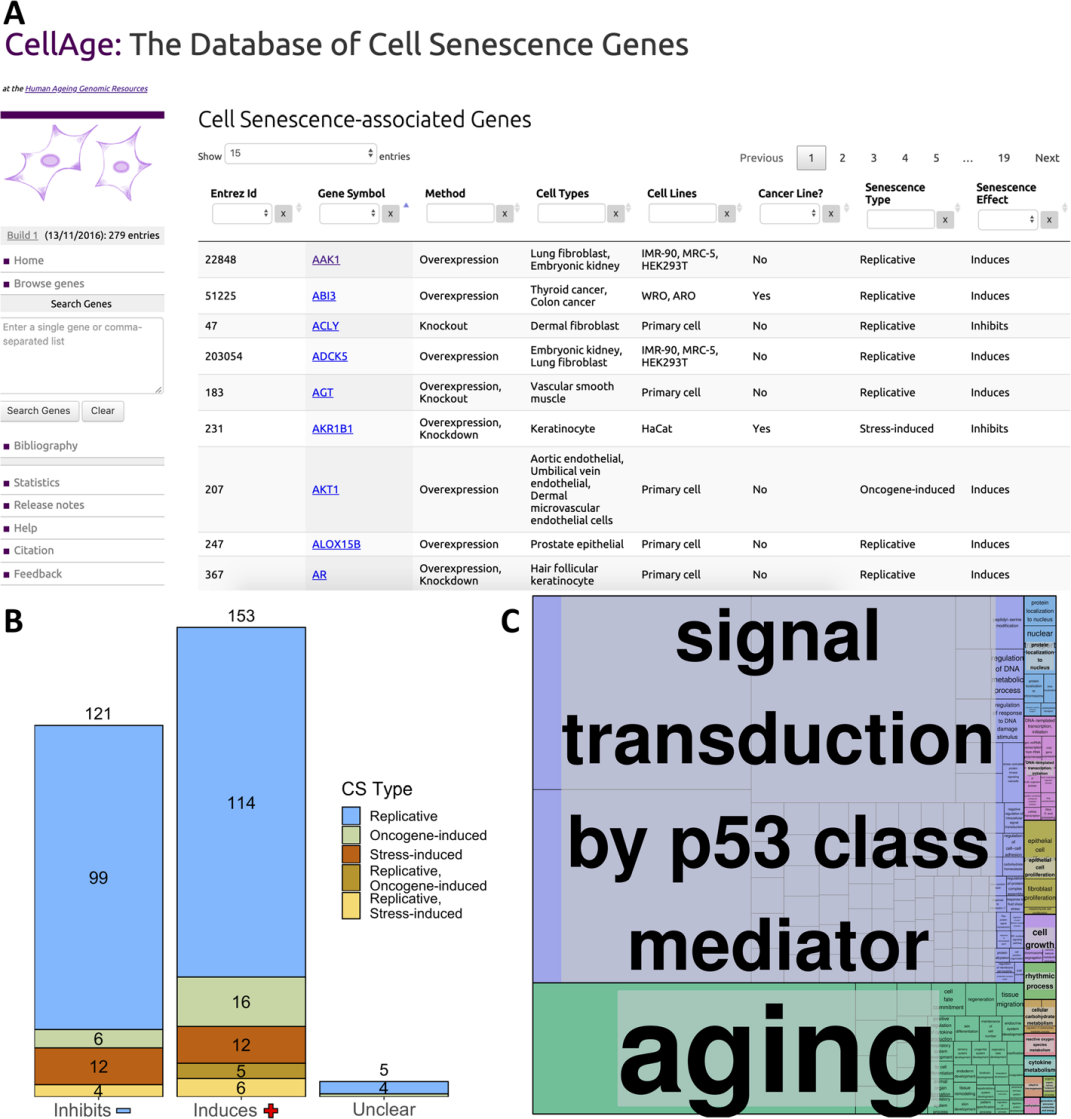
**a** The CellAge database of CS genes. The main data browser provides functionality to filter by multiple parameters like cell line and senescence type, and select genes to view details and links with other aging-related genes on the HAGR website. **b** Breakdown of the effects all 279 CellAge genes have on CS, and the types of CS the CellAge genes are involved in. Genes marked as “Unclear” both induce and inhibit CS depending on biological context. Numbers above bars denote the total number of genes inhibiting, inducing, or having unclear effects on CS. **c** Functional enrichment of the nonredundant biological processes involving the CellAge genes (*p* < 0.05, Fisher’s exact test with BH correction) (Additional file 1: Table S3). GO terms were clustered based on semantic similarities

The CellAge website can be accessed at http://genomics.senescence.info/cells/. Figure 1a presents the main CellAge data browser, which allows users to surf through the available data. The browser includes several columns with information that can be searched and filtered efficiently. Users can search for a comma-separated gene list or for individual genes. Once selected, a gene entry page with more detailed description of the experimental context will open.

CellAge was compiled following a scientific literature search of gene manipulation experiments in primary, immortalized, or cancer human cell lines that caused cells to induce or inhibit CS. The first CellAge build comprises 279 distinct CS genes, of which 232 genes affect replicative CS, 34 genes affect stress-induced CS, and 28 genes affect oncogene-induced CS. Of the 279 total genes, 153 genes induce CS (~ 54.8%), 121 inhibit it (~ 43.4%), and five genes have unclear effects, both inducing and inhibiting CS depending on experimental conditions (~ 1.8%) (Fig. 1b). The genes in the dataset are also classified according to the experimental context used to determine these associations. 

We have also performed a meta-analysis to derive a molecular signature of replicative CS and found 526 overexpressed and 734 underexpressed genes [32]. These gene signatures are also available on the CellAge website. Of the 279 CellAge genes, 44 genes were present in the signatures of CS (15.8%). This overlap was significant (*p* value = 1.62e−08, Fisher’s exact test). While 13 of the CellAge inducers of CS significantly overlapped with the overexpressed signatures of CS (8.5%, *p* = 2.06e−06, Fisher’s exact test), only 7 overlapped with the underexpressed signatures (4.6%, *p* = 5.13e−01, Fisher’s exact test). The CellAge inhibitors of CS significantly overlapped with both the overexpressed signatures of CS (*n* = 7, 5.8%, *p* = 4.08e−02, Fisher’s exact test) and underexpressed signatures of CS (*n* = 17, 14%, *p* = 2.06e−06, Fisher’s exact test).

### CellAge gene functions

High-quality curated datasets enable systematic computational analyses [33, 34]. Since we are interested in learning more about the underlying processes and functionality shared by human CS genes, we started by exploring functional enrichment within the CellAge dataset.

Using the database for annotation, visualization and integrated discovery—DAVID Version 6.8 [35, 36], we found that genes in CellAge are enriched with several clusters associated with Protein Kinase Activity, Transcription Regulation, DNA-binding, DNA damage repair, and Cell cycle regulation in cancer. In particular, genes that induce senescence were more associated with promoting transcription, while genes that inhibit senescence were more associated with repressing transcription. Furthermore, we found that inducers of senescence were significantly associated with VEGF and TNF signalling pathways (*p* < 0.01, Fisher’s exact test with Benjamini-Hochberg correction) (Additional file 1: Table S1 and S2). WebGestalt 2019 was used to determine which nonredundant biological processes the CellAge genes are involved in, and REVIGO was used to cluster related processes (*p* < 0.05, Fisher’s exact test with BH correction) [37, 38]. A total of 298 categories were significantly enriched and clustered: Signal transduction by p53 class mediator; Aging; Protein localization to nucleus; DNA-templated transcription, initiation; Epithelial cell proliferation; Cell growth; Rhythmic process; Cellular carbohydrate metabolism; Reactive oxygen species metabolism; Cytokine metabolism; Adaptive thermogenesis; Organic hydroxy compound metabolism; Methylation; Generation of precursor metabolites and energy (Fig. 1c; Additional file 1: Table S3).

### Evolutionary conservation of CellAge genes in model organisms

Next, we looked at the conservation of CellAge genes across a number of mammalian and non-mammalian model organisms with orthologues to human CellAge genes using Ensembl BioMart (Version 96) [39] in order to understand the genetic conservation of CS processes. There was a significantly higher number of human orthologues for CellAge genes than for other protein-coding genes in mouse, rat, and monkey, while non-mammalian species did not show significant conservation of CellAge genes (two-tailed *z*-test with BH correction) (Additional file 1: Table S4; Additional file 2: Fig. S1A). Interestingly, previous studies have found that longevity-associated genes (LAGs) are substantially overrepresented from bacteria to mammals and that the effect of LAG overexpression in different model organisms was mostly the same [40]. It remains unclear what the evolutionary origin of most of the CellAge genes is or why they are not present in more evolutionarily distant organisms. Unique evolutionary pressures could have played an important role in the evolution of CellAge genes in mammals. However, somatic cells in *C. elegans* and *Drosophila* are post mitotic and lack an equivalent CS process, which could explain why the CellAge genes are not conserved. We further compared the conservation of CellAge inducers and inhibitors of CS and found that while the inducers were significantly conserved in the mammal model organisms, the inhibitors were not (Additional file 2: Fig. S1B).

We also report the number of orthologous CellAge genes present in 24 mammal species using the OMA standalone software v. 2.3.1 algorithm [41] (Additional file 2: Fig. S1C). From 279 CellAge genes, we report 271 orthogroups (OGs) (Additional file 3). Twenty-two OGs were conserved in the 24 mammals, including the following genes: *DEK*, *BRD7*, *NEK4*, *POT1*, *SGK1*, *TLR3*, *CHEK1*, *CIP2A*, *EWSR1*, *HDAC1*, *HMGB1*, *KDM4A*, *KDM5B*, *LATS1*, *MORC3*, *NR2E1*, *PTTG1*, *RAD21*, *NFE2L2*, *PDCD10*, *PIK3C2A*, and *SLC16A7* (Additional file 1: Table S5). Within the long-lived mammalian genomes analyzed (human, elephant, naked mole rat, bowhead whale, and little brown bat), we found 128 OG CellAge genes (Additional file 3; genomes available in Additional file 1: Table S6). However, finding OGs is dependent on genome quality and annotations, and higher-quality genomes would likely yield more OGs.

For the evolutionary distances, we found that the long-lived species had similar distances to the other species, meaning the branch lengths for long-lived species are distributed throughout the phylogeny as expected in a random distribution (Additional file 2: Fig. S1D). This was the case when we analyzed the concatenated tree for the 271 CellAge OGs as well as when we analyzed the 22 individual CellAge genes conserved among all 24 mammalian species (Additional file 4).

### CellAge vs human orthologues of longevity-associated model organism genes

To understand how senescence is linked to the genetics of aging processes, we looked at the intersection of CellAge genes and the 869 genes in the human orthologues of model organisms’ longevity-associated genes (LAGs) dataset, collected based on quantitative changes in lifespan [34]. Like CellAge, where genes are classified based on whether their upregulation induces, inhibits, or has an unknown impact on CS, the longevity orthologues dataset also provides information on the effect of upregulation of its genes, namely whether it promotes (pro, 421) or inhibits (anti, 448) longevity (Additional file 1: Table S7; Additional file 2: Fig. S2).

The CS inducers statistically overlapped with the anti-longevity genes and not with the pro-longevity genes (anti: *n* = 9, ~ 6%, *p* = 1.42e−02; pro: *n* = 6, ~ 4%, *p* = 1.40e−01, Fisher’s exact test with BH correction). We noted an inverse result with the inhibitors of CS, where there was a much greater overlap between the CellAge inhibitors and the pro-longevity genes, resulting in the smallest *p* value of all the overlaps (*n* = 18, ~ 15%, *p* = 2.61e−10, Fisher’s exact test with BH correction). However, there was also a significant overrepresentation of genes inhibiting the CS process within the anti-longevity genes (*n* = 7, ~ 6%, *p* = 2.41e−02, Fisher’s exact test with BH correction). It is possible that some of the pathways the CS inhibitors are associated with increase longevity, whereas other pathways have anti-longevity effects. Overall, these results highlight a statistically significant association between CS and the aging process and suggest a potential inverse relationship between CS and longevity, at least for some pathways. Gene overlaps are available in Additional file 1: Table S8.

### CellAge genes differentially expressed with age

In another work, we performed a meta-analysis to find molecular signatures of aging derived from humans, rats, and mice [42]. To investigate how the expression of CellAge genes changes with age, we looked for CellAge genes which either induce (153) or inhibit (121) senescence within the list of aging signatures. The genes overexpressed with age (449) had a significant overlap with the CellAge genes (CS inducers: *n* = 17, ~ 11%, *p* = 6.58e−07; CS inhibitors: *n* = 9, ~ 7%, *p* = 6.35e−03, two-tailed Fisher’s exact test with BH correction) while the genes underexpressed with age (162) did not (CS inducers: *n* = 0, *p* = 8.57e−01; CS inhibitors: *n* = 3, ~ 3%, *p* = 1.64e−01). The overexpressed genetic signatures of replicative CS (526) also significantly overlapped with the overexpressed signatures of aging (*n* = 60, ~ 11%, *p* = 1.18e−23), but not the underexpressed signatures of aging (*n* = 3, ~ 1%, *p* = 8.79e−01). Finally, the underexpressed signatures of replicative CS (734) did not significantly overlap with the overexpressed (*n* = 18, ~ 3%, *p* = 8.79e−01) or underexpressed (*n* = 9, ~ 1%, *p* = 3.26e−01) signatures of aging.

Given that 112 (40%) of CellAge genes have only been confirmed to control CS in fibroblasts, we repeated the above analyses using a subgroup of CellAge genes that have been shown to affect CS in other cell types. A total of 91 CellAge inducers of CS and 72 inhibitors were overlapped with the signatures of aging. The same overlaps were still significant after FDR correction, indicating that the differential expression of CellAge genes with age cannot exclusively be attributed to fibroblast idiosyncrasies (CS inducers overexpressed: *n* = 10, ~ 11%, *p* = 1.50e−04; underexpressed: *n* = 0, *p* = 1. CS inhibitors overexpressed: *n* = 6, ~ 8%, 1.34e−02; underexpressed: *n* = 2, ~ 3%, *p* = 1.98e−01).

Using all protein-coding genes from the meta-analysis as a background list [42], we further examined the CS inducers overexpressed with age for functional enrichment using WebGestalt 2019 to determine if specific biological processes were enriched [38]. In parallel, we performed this analysis using the genes which overlapped between CellAge inhibitors and genes overexpressed with age. In total, 71 GO terms were significantly enriched for the overlap between CellAge senescence inducers and age upregulated genes (*p* < 0.05 Fisher’s exact test with BH correction) (Additional file 1: Table S9). Because many of the enriched GO terms were redundant (e.g., wound healing and response to wound healing, regulation of cytokine production and cytokine production), they were clustered based on semantic similarity scores using REVIGO [37]. We found groups enriched for regulation of apoptotic processes, response to lipid, epithelium development, rhythmic process, circadian rhythm, cytokine metabolism, and cell-substrate adhesion (Additional file 2: Fig. S3A). A total of 71 enriched GO terms for the overexpressed signatures of CS overexpressed with age were clustered using REVIGO, resulting in enriched terms relating to regulated exocytosis, aging, response to beta-amyloid, and cell proliferation (Additional file 1: Table S10; Additional file 2: Fig. S3B). No GO terms were significantly enriched for the inducers of CS underexpressed with age, the inhibitors of CS differentially expressed with age, the underexpressed signatures of CS differentially expressed with age, or the overexpressed signatures of CS underexpressed with age.

### Tissue-specific CS gene expression and differential expression of CS genes in human tissues with age

The Genotype-Tissue Expression (GTEx) project contains expression data from 53 different tissue sites collected from 714 donors ranging from 20 to 79 years of age, grouped into 26 tissue classes [43]. We asked if CellAge genes and differentially expressed signatures of CS were expressed in a tissue-specific manner [42] and determined how CS gene expression changes across different tissues with age [32].

We first examined tissue-specific CS expression and found that CellAge genes were either expressed in a tissue-specific manner less than expected by chance, or in line with expectations; in other words, the majority of CellAge genes tended to be expressed across multiple tissues (Additional file 1: Table S11; Additional file 2: Fig. S4A). Testis was the only tissue with significant differences between the actual and expected number of tissue-specific CellAge genes expressed (less tissue-specific genes than expected by chance, *p* < 0.05, Fisher’s exact test with BH correction). The underexpressed signatures of CS were significantly less tissue-specific in the testis and liver, while the overexpressed signatures of CS were significantly less tissue-specific in the brain, liver, pituitary, and skin, and more tissue-specific in blood. We also compared the ratio of tissue-specific to non-tissue-specific genes in the CS datasets to all protein-coding genes. While ~ 25% of all protein-coding genes are expressed in a tissue-specific manner, only ~ 10% of CellAge genes and ~ 11% of signatures of CS are expressed in a tissue-specific manner (Additional file 2: Fig. S4B), significantly less than expected by chance (*p* = 2.52e−12 and 3.93e−48 respectively, Fisher’s exact test with BH correction).

**Fig. 2:**
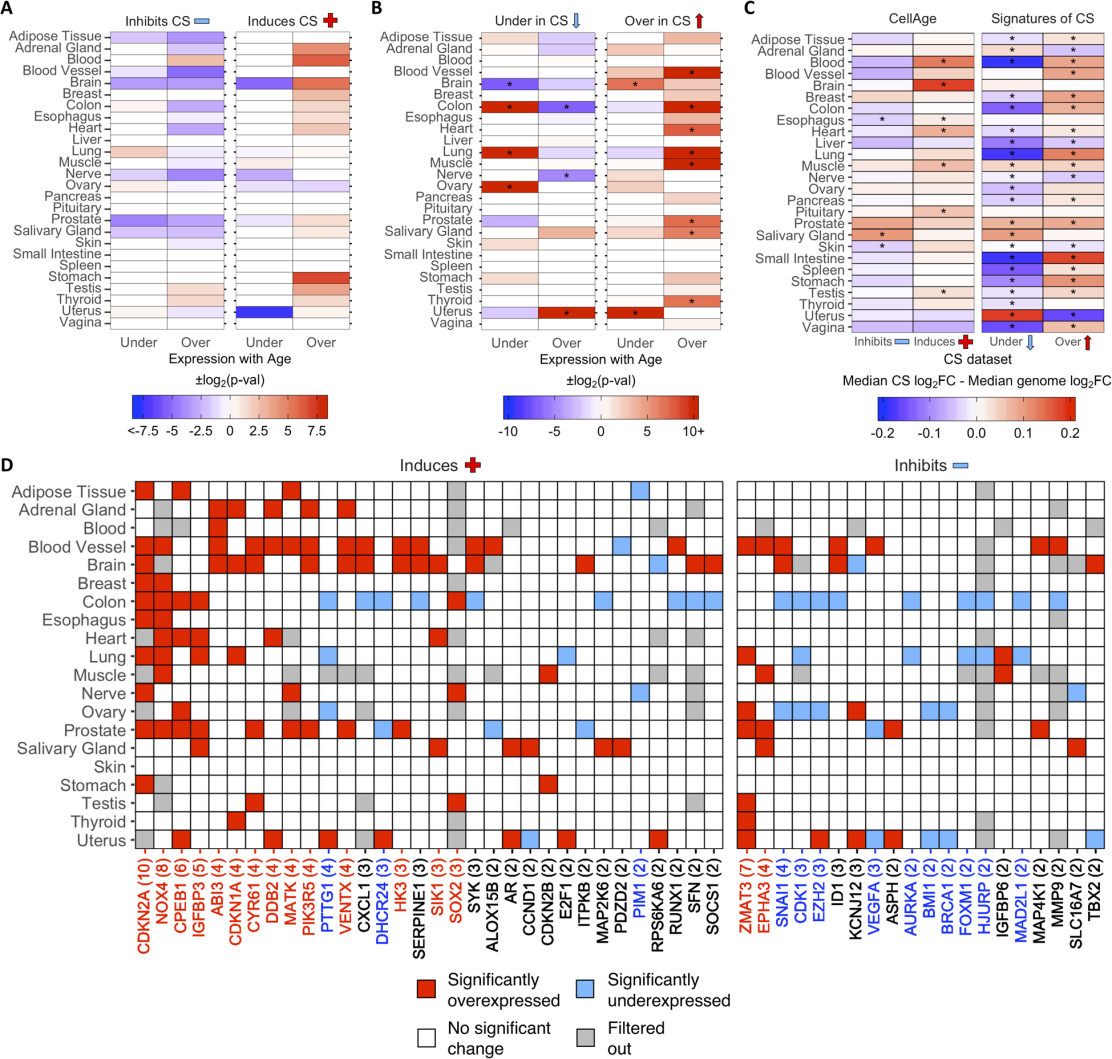
Differential expression of **a** CellAge inducers and inhibitors of CS and **b** differentially expressed signatures of CS in human tissues with age. Red values indicate that there were more genes differentially expressed with age than expected by chance (−log_2_(*p*-val)). Blue values indicate that there were less genes differentially expressed with age than expected by chance (log_2_(*p*-val)). Asterisks (*) denote tissues with significantly more CS genes differentially expressed with age (*p* < 0.05, Fisher’s exact test with BH correction, abs(50*log_2_FC) > log_2_(1.5)) (Additional file 1: Table S12 and S13). **c** Comparison of the median log_2_FC and distribution of log_2_FC with age between the CS genes and all protein-coding genes in human tissues. Red tiles indicate that the median log_2_FC of the CellAge and CS genes is higher than the median log_2_FC of all protein-coding genes for that tissue, while blue tiles indicate that the median log_2_FC of the CS genes is lower than the median genome log_2_FC. Asterisks (*) indicate significant differences between the log_2_FC distribution with age of CS genes and the log_2_FC distribution with age of all protein-coding genes for that tissue (*p* < 0.05, Wilcoxon rank sum test with BH correction) (Additional file 1: Table S16). **d** CellAge genes differentially expressed in at least two tissues with age. Gray tiles are genes which had low basal expression levels in the given tissue and were filtered out before the differential gene expression analysis was carried out [32]. Colored tiles indicate significant differential expression with age (*p* < 0.05, moderated *t*-test with BH correction, abs(50*log_2_FC) > log_2_(1.5)). Numbers by gene names in brackets denote the number of tissues differentially expressing the CellAge gene with age. Red gene names specify that the CellAge gene was significantly overexpressed with age in more tissues than expected by chance, while blue gene names show the CellAge genes significantly underexpressed with age in more tissues than expected by chance (*p* < 0.05, random gene expression tissue overlap simulations) (Additional file 1: Table S17 – S20). Liver, pancreas, pituitary, spleen, small intestine, and vagina did not have any significant CS DEGs with age

Then, we examined the differential expression of CS genes with age in different tissues. Using a previously generated gene set of differentially expressed genes (DEGs) with age in 26 tissues on GTEx [32, 43], we found overlaps with 268 CellAge inducers and inhibitors of CS present in the gene expression data (Fig. 2a). The process of finding DEGs with age filters out lowly expressed genes, which explains the 11 missing CellAge CS regulators. Overall, senescence inducers were overexpressed across different tissues with age, although none of the overlaps were significant after FDR correction (Fisher’s exact test with BH correction, *p* < 0.05) (Additional file 1: Table S12). There was the opposite trend in the inhibitors of CS, where there was noticeably less overexpression of CS inhibitors with age, although these overlaps were also not significant after FDR correction. A total of 1240 differentially expressed signatures of CS were also overlapped with the GTEx aging DEGs in 26 human tissues, including 9 tissues previously analyzed (Fig. 2b) [32]. The overexpressed signatures of CS were significantly overexpressed across multiple tissues with age, and only significantly underexpressed with age in the brain and uterus (*p* < 0.05, Fisher’s exact test with BH correction) (Additional file 1: Table S13). Furthermore, the underexpressed signatures of CS trended towards being overexpressed less than expected by chance across multiple tissues with age, although these overlaps were only significant after FDR adjustment in the colon and nerve, while the underexpressed signatures of CS were significantly overexpressed more than expected in the uterus. Finally, the underexpressed signatures of CS were underexpressed with age more than expected by chance in the colon, lung, and ovary, and underexpressed with age less than expected by chance in the brain. We also compared the ratio of differentially expressed to non-differentially expressed CS genes in at least one tissue with age to the equivalent ratio in all protein-coding genes (Additional file 2: Fig. S5A and S5B) (see Overlap Analysis in Methods). We found that ~ 64% of all protein-coding genes did not significantly change expression with age in any human tissues, while ~ 19% were overexpressed and ~ 17% were underexpressed (~ 7% were both overexpressed and underexpressed across multiple tissues) (Additional file 1: Table S14 and S15). For the CellAge genes, the number of inducers of CS significantly overexpressed with age in at least one tissue was significantly higher than the genome average (*n* = 50, ~ 30%, *p* = 1.5e−3, Fisher’s exact test with BH correction). The inducers of CS underexpressed with age and the inhibitors of CS differentially expressed with age were not significantly different from the protein-coding average. We also compared the number of signatures of CS differentially expressed with age in at least one tissue to the protein-coding genome average. The overexpressed signatures of CS were significantly differentially expressed with age compared to all protein-coding genes, whereas the number of underexpressed signatures of CS was underexpressed with age more than expected by chance.

The overall fold change (FC) with age of the CS genes was also compared to the FC with age of all protein-coding genes for each tissue in GTEx (Fig. 2c; Additional file 1: Table S16). The median log_2_FC with age of the CellAge CS inducers and the overexpressed signatures of CS was greater than the genome median for the majority of tissues on GTEx, although the difference in log_2_FC distribution with age between the inducers of CS and all protein-coding genes was only significant in seven tissues (Wilcoxon rank sum test with BH correction, *p* < 0.05). The median log_2_FC with age of the CellAge inhibitors of CS and the underexpressed signatures of aging was smaller than the genome median in the majority of tissues, showcasing the opposite trend to the inducers of CS and overexpressed signatures of CS. However, the only tissues with significantly different distributions of log_2_FC with age for the inhibitors of CS were the skin and esophagus, where the median log_2_FC distribution was significantly less than the genome average, and the salivary gland, where the median log_2_FC distribution was significantly more than the genome average. We also found that the distribution of log_2_FC with age of the differentially expressed signatures of CS significantly changed in opposite directions with age in 14 tissues. Interestingly, this trend was present even in the adrenal gland and uterus, where the signatures of CS changed with age in the opposite direction to the majority of other tissues.

The expression of the majority of CS genes does not change with age (Additional file 2: Fig. S5A), yet a significant number of CS genes trend towards differential expression with age across multiple tissues in humans (Fig. 2). We ran 10,000 simulations on the GTEx RNA-seq data to determine the likelihood of a CS gene being differentially expressed with age in more than one tissue by chance (see Simulation of CS Gene Expression in Human Aging in Methods) (Additional file 2: Fig. S5C; Additional file 5). The likelihood of a CellAge gene being overexpressed with age in more than three tissues and underexpressed with age in more than two tissues by chance was less than 5% (CS gene expression simulations) (Fig. 2d; Additional file 1: Table S17; Additional file 2: Fig. S5C). CS inducers overexpressed in significantly more tissues with age than expected by chance included *CDKN2A*, *NOX4*, *CPEB1*, *IGFBP3*. *ABI3*, *CDKN1A*, *CYR61*, *DDB2*, *MATK*, *PIK3R5*, *VENTX*, *HK3*, *SIK1*, and *SOX2*, while *PTTG1*, *DHCR24*, *IL8*, and *PIM1* were underexpressed in significantly more tissues (Additional file 1: Table S18; Additional file 2: Fig. S5D). *ZMAT3* and *EPHA3* were the two CS inhibitors overexpressed in significantly more tissues with age than expected by chance, while *CDK1*, *AURKA*, *BMI1*, *BRCA1*, *EZH2*, *FOXM1*, *HJURP*, *MAD2L1*, *SNAI1*, and *VEGFA* were underexpressed in significantly more tissues. We also performed simulations to determine the likelihood of gene expression signatures of CS being differentially expressed with age in multiple human tissues by chance (Additional file 1: Table S19): less than 5% of the genes in the CS signatures are expected by chance to be overexpressed with age in more than three tissues or underexpressed with age in more than two tissues. A total of 46 CS signature genes (29 overexpressed, 17 underexpressed) were overexpressed with age in significantly more tissues than expected by chance, and 139 CS signature genes were underexpressed in more tissues than expected by chance (26 overexpressed genes in CS, 113 underexpressed genes in CS) (Additional file 1: Table S20).

### Do CS and longevity genes associate with aging-related disease genes?

A previous paper [34] grouped 769 aging-related diseases (ARDs) into 6 NIH Medical Subject Heading (MeSH) classes [44] based on data from the Genetic Association Database [45]: cardiovascular diseases (CVD), immune system diseases (ISD), musculoskeletal diseases (MSD), nutritional and metabolic diseases (NMD), neoplastic diseases (NPD), and nervous system diseases (NSD). The same approach was used to build the HAGR aging-related disease gene selection tool (http://genomics.senescence.info/diseases/gene_set.php), which we used to obtain the ARD genes for each disease class and overlap with the CellAge genes.

There were links between the CellAge genes and NPD genes, which is expected given the anti-tumor role of senescence (Additional file 1: Table S21). Without accounting for publication bias (i.e., some genes being more studied than others), all ARD classes are significantly associated with CellAge genes, with lower commonalities with diseases affecting mostly non-proliferating tissue such as NSD. NPD genes are even more overrepresented in the GenAge human dataset, which could suggest commonality between aging and senescence through cancer-related pathways. Both the strong association of NPD genes with GenAge and senescence, and the strong link between GenAge and all ARD classes is interesting. Indeed, longevity-associated genes have been linked to cancer-associated genes in previous papers [46]. Considering age is the leading risk factor for ARD [47, 48], the results from GenAge support the previously tested conjecture that there are (i) at least a few genes shared by all or most ARD classes; and (ii) those genes are also related to aging in general [34]. We also looked for genes that are shared across multiple disease classes and are also recorded as CS genes. CellAge genes shared across multiple ARD classes included *VEGFA* and *IFNG* (5 ARD classes), *SERPINE1*, *MMP9*, and *AR* (4 ARD classes), and *CDKN2A* (3 ARD classes). Results are summarized in Additional file 2: Fig. S6.

### Are CS genes associated with cancer genes?

**Fig. 3:**
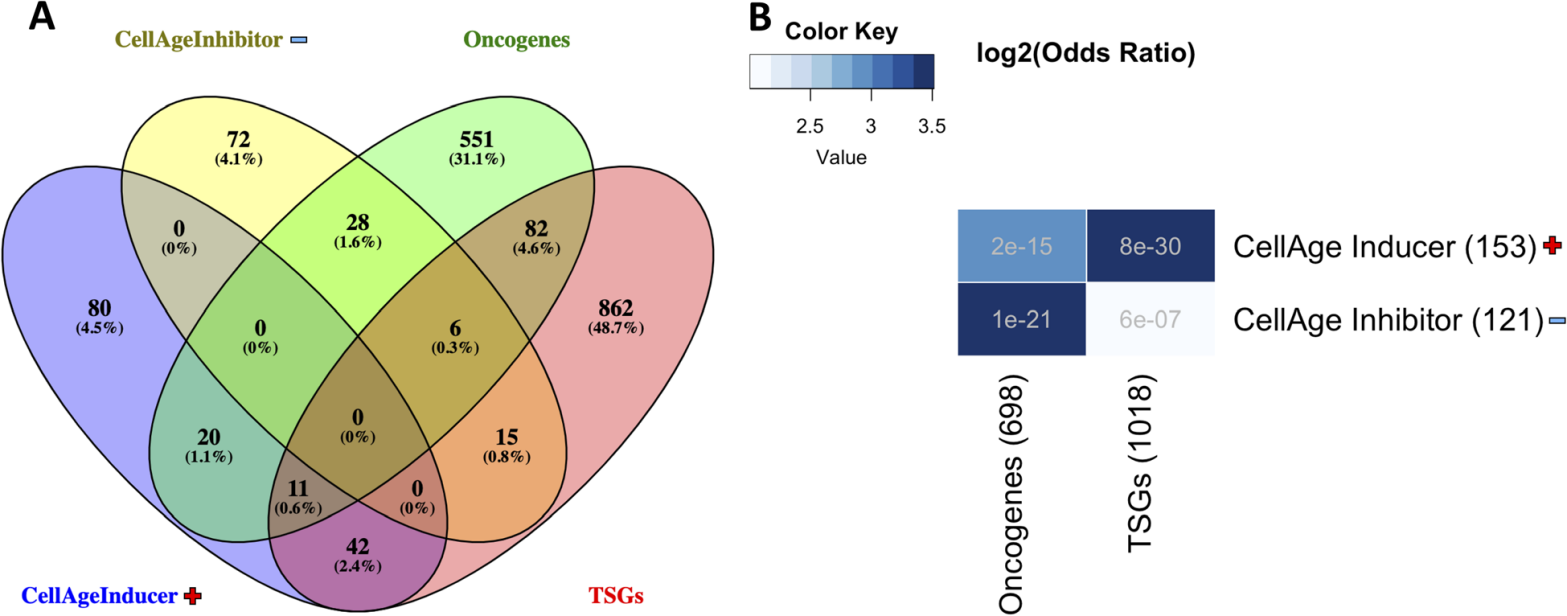
**a** Overlap between CellAge inducers and inhibitors, and oncogenes and tumor-suppressing genes. **b** Adjusted *p* value and odds ratio of the overlap analysis. The number of overlapping genes in each category was significant (*p* < 0.05, Fisher’s exact test with BH correction). *p* values are shown in gray writing for each comparison. Data available in Additional file 1: Table S22 – S27

Cellular senescence is widely thought to be an anti-cancer mechanism [49]. Therefore, the CellAge senescence inducers and inhibitors of senescence were overlapped with oncogenes from the tumor-suppressor gene (TSG) database (TSGene 2.0) (*n* = 1018) [50] and the ONGene database (*n* = 698) [51] (Additional file 1: Table S22 – S27). The number of significant genes overlapping are shown in Fig. 3a, while the significant *p* values from the overlap analysis are shown in Fig. 3b (*p* < 0.05, Fisher’s exact test with BH correction).

The significant overlap between CellAge genes and cancer indicates a close relationship between both processes. Specifically, the overlap between CellAge inhibitors and oncogenes, and the overlap between CellAge inducers and TSGs were more significant, with lower *p* values and larger odds ratios (Fig. 3) [52]. This analysis was repeated after filtering out CellAge genes that were only shown to induce senescence in fibroblasts. The overlaps were still significant after FDR correction, indicating that the overlap between CellAge and cancer genes is not specific to genes controlling CS in fibroblasts (CS inducers with oncogenes: *n* = 10, *p* = 9e−05; with TSGs: *n* = 23, *p* = 4e−12. CS inhibitors with oncogenes: *n* = 17, 1e−12; with TSGs: *n* = 8, *p* = 9e−04, *p* < 0.05, Fisher’s exact test with BH correction) (Additional file 2: Fig. S7).

Gene ontology (GO) enrichment analyses were performed using WebGestalt to identify the function of the overlapping genes [38]. Overlapping genes between CellAge senescence inducers and TSGs were enriched in GO terms related to p53 signalling and cell cycle phase transition (Additional file 2: Fig. S8A). The enriched functions of overlapping genes between CellAge senescence inducers and oncogenes were mainly related to immune system processes and response to stress (Additional file 2: Fig. S8B). Overlapping genes between CellAge senescence inhibitors and TSGs were enriched in only 5 terms, which are cellular response to oxygen-containing compound, positive regulation of chromatin organization, and terms relating to female sex differentiation (Additional file 2: Fig. S8C). Finally, overlapping genes between CellAge senescence inhibitors and oncogenes were related to processes such as negative regulation of nucleic acid-templated transcription, cellular response to stress, and cell proliferation (Additional file 2: Fig. S8D). All of the functional enrichment data can be found in Additional file 1: Table S28 – S31.

### Network analyses

The CellAge genes form both protein-protein and gene co-expression networks. The formation of a protein-protein interaction (PPI) network is significant in itself given that only ~ 4% of the genes in a randomly chosen gene dataset of similar size are interconnected [53]. In order to have a more holistic view of CS, we were interested in the topological parameters of the networks that CS genes form. For this, several types of networks were constructed using the CellAge genes as seeds: the CS PPI network, along with two CS gene co-expression networks built using RNA-seq and microarray data. Biological networks generally have a scale-free topology in which the majority of genes (nodes) have few interactions (edges), while some have many more interactions, resulting in a power law distribution of the node degree (the number of interactions per node) [31, 54]. As expected, the node-degree distribution of the above networks does confirm a scale-free structure (Additional file 2: Fig. S9). Additional file 1: Table S32 presents the network summary statistics for the resulting networks.

The network parameters we looked at were as follows: Degree, Betweenness Centrality (BC), Closeness Centrality (CC), and Increased Connectivity (IC). The degree is the number of interactions per node and nodes with high degree scores are termed network hubs. BC is a measure of the proportion of shortest paths between all node pairs in the network that cross the node in question. The nodes with high BC are network bottlenecks and may connect large portions of the network which would not otherwise communicate effectively or may monitor information flow from disparate regions in the network [31]. CC is a measure of how close a certain node is to all other nodes and is calculated with the inverse of the sum of the shortest paths to all other nodes. Lower CC scores indicate that nodes are more central to the network, while high CC scores indicate the node may be on the periphery of the network and thus less central. The IC for each node measures the statistical significance for any overrepresentation of interactions between a given node and a specific subset of nodes (in our case CellAge proteins) when compared to what is expected by chance. Taken together, genes that score highly for degree, BC, CC, and IC within the senescence networks are likely important regulators of CS even if up until now they have not been identified as CS genes.

Looking at the topology of CS networks, the PPI network, microarray-based co-expression network, and RNA-seq co-expression network all possess comparable scale-free structures. However, gene co-expression data is less influenced by publication bias. This is particularly important considering published literature often reports positive protein-protein interactions over protein interactions that do not exist [55]. The lack of negative results for protein interaction publications complicates the interpretation of PPI networks even more, as the absence of edges in networks does not necessarily mean they do not exist. On the other hand, RNA-seq and microarray co-expression data, while not influenced by publication bias, does not give indications of actual experimentally demonstrated interactions (physical or genetic). Furthermore, RNA read counts do not directly correlate to protein numbers, with previous studies reporting that only 40% of the variation in protein concentration can be attributed to mRNA levels, an important aspect to consider when interpreting RNA-seq data [56]. Finally, the microarray network was constructed using the COXPRESdb (V6), which contains 73,083 human samples and offered another degree of validation [57]. Although RNA-seq reportedly detects more DEGs including ncRNAs [58], GeneFriends [59] contains 4133 human samples, far less than the microarray database from COXPRESdb.

#### The protein-protein interaction network associated with CS

We only used interactions from human proteins to build the CellAge PPI network. The network was built by taking the CellAge genes, their first-order partners and the interactions between them from the BioGrid database. The CellAge PPI network comprised of 2487 nodes across four disjointed components, three of which only comprised of two nodes each, and the main component containing 2481 nodes.

The genes with the highest degree scores were *TP53*, *HDAC1*, *BRCA1*, *EP300,* and *MDM2*. These same genes also ranked in the top five CC. Expectedly, several of these genes also possessed the highest BC: *TP53*, *BRCA1, HDAC1*, and *MDM2* (with *BAG3*, a gene with a slightly smaller degree also within the top 5). On the other hand, the genes ranked by top 5 IC were *CCND1*, *CCND2, CDKN2A, SP1,* and *EGR1*. Of note among these nodes, *EP300*, *MDM2*, *CCND2,* and *EGR1* were not already present in CellAge. Additional file 2: Fig. S10 summarizes the gene intersection across the computed network parameters, while Additional file 1: Table S33 identifies potential senescence regulators not already present in CellAge from the PPI network. We found that from the top 12 PPI candidates, 11 have been recently shown to regulate senescence in human cell lines and will be added to CellAge build 2.

Within the main PPI network component, a large portion of CS genes and their partners formed a single large module with 1595 nodes. Using DAVID version 6.8, we found the terms enriched within the module; the top five are: Transcription, DNA damage & repair, cell cycle, Proteasome & ubiquitin, and ATP pathway [35, 36] (Additional file 1: Table S34). These results are all in line with previously described hallmarks of cellular senescence [60].

It is prudent to note that centrality measures in PPI networks must be interpreted with caution due to publication bias that can be an inherent part of the network [61, 62]. The top network genes identified from the PPI network are likely to be heavily influenced by publication bias [63]. Looking at the average PubMed hits of the gene symbol in the title or abstract revealed a mean result count of approximately 2897 per gene, far higher than the genome average (136) or existing CellAge genes (712) (Additional file 2: Fig. S11).

#### Unweighted RNA-Seq co-expression network

**Fig. 4:**
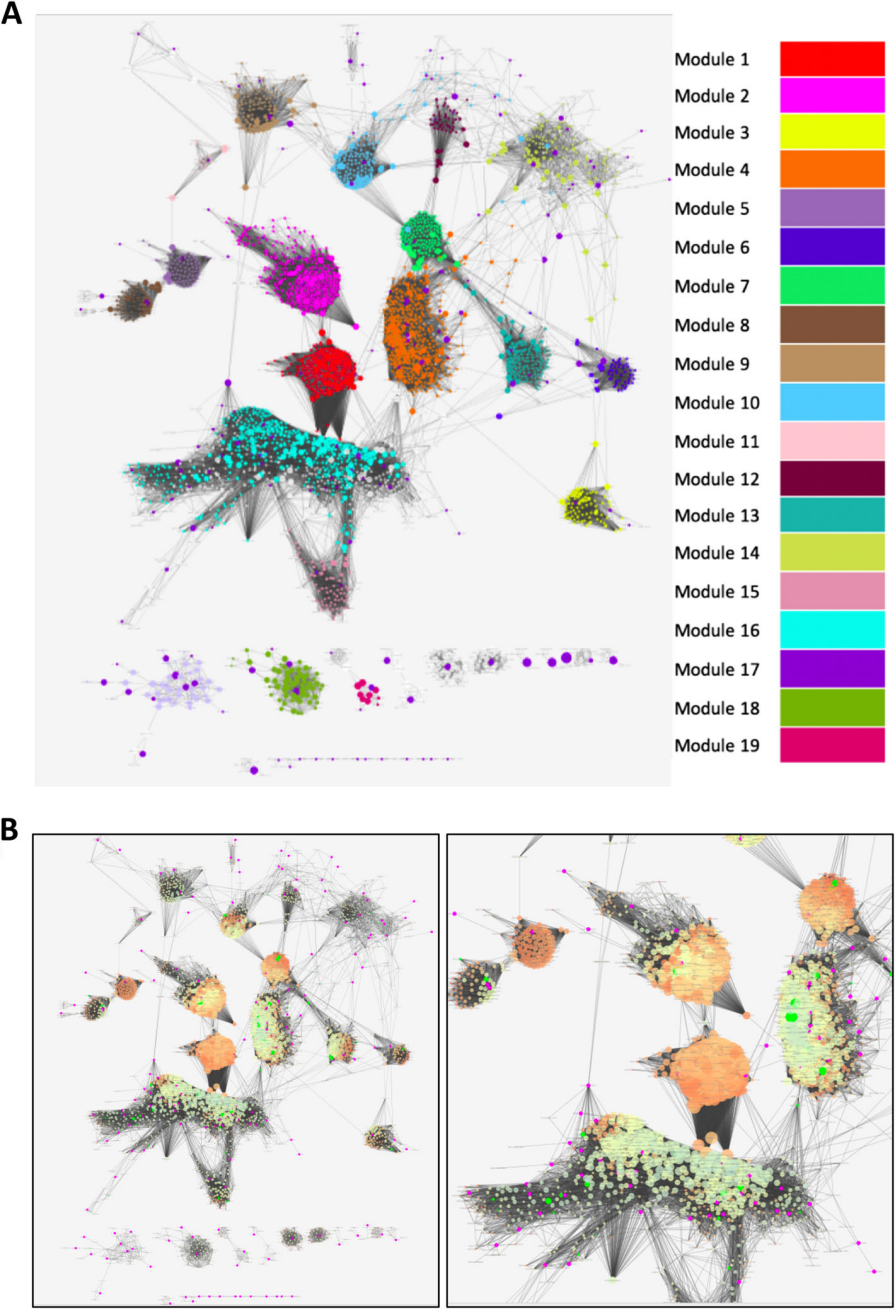
**a** Cluster analysis of the RNA-Seq Unweighted Co-expression Network. The 171 seed nodes obtained from CellAge and their first order interactors. The colours represent the breakdown of the network into clusters. The algorithm revealed 52 distinct clusters, of which we color and order the 19 clusters with the best rankings for modularity, or in the case of module 17–19, size. The CellAge nodes are colored in dark purple, appearing throughout the network. Larger nodes have higher betweenness centrality. In order of decreasing modularity, the main function clusters of the modules were related to; Spermatogenesis (Module 1), Synapse (Module 2), Cardiac muscle contraction (Module 3), Cell Cycle (Module 4), Secreted (Module 5), Tudor domain (Module 6), ATP-binding (Module 7), Symport (Sodium ion transport) (Module 8), DNA damage and repair (Module 9), transit peptide: Mitochondrion (Module 10), Steroid metabolism (Module 11), Transcription regulation (Module 12), Protein transport (Module 13), Mitochondrion (Module 14), Heme biosynthesis (Module 15), Innate immunity (Module 16), Signal peptide (Module 17), Keratinocyte (Module 18), and Transcription repression (Module 19) (Enrichment results in Additional file 1: Table S35, genes in Additional file 1: Table S36). **b** RNA-Seq Unweighted Co-expression Network, local clustering. Red/Orange represents nodes with high clustering coefficient, whereas pale green represents nodes with lower clustering coefficient. Degree is also weighted using node size. CellAge nodes are colored purple, and GenAge Human nodes are also shown and highlighted in bright green. The right-hand panel is an enlarged view of the left-hand panel

We used CellAge genes that induce and inhibit CS and their co-expressing partners to build a cellular senescence co-expression network. The network consists of a main connected network with 3198 nodes, and a number of smaller “islands” that are not connected to the main network (Fig. 4a).

The main interconnected network included 130 CellAge genes. Among these, we also found that 14% of them are also human aging-related genes, reported in GenAge - Human dataset, whereas the remainder of the smaller networks only comprised of 1.6% longevity genes [64]. Next, we looked at a number of centrality parameters to see how CellAge genes are characterized compared to the entire network. CellAge genes had a mean BC of 0.00363, whereas the remainder of the genes had a BC of 0.00178, revealing that if CellAge genes are removed, modules within the network may become disconnected more easily. While nodes scoring highly for BC in PPI networks are likely bottleneck regulators of gene expression, this is not necessarily true for co-expression networks. In this case, nodes can also have high BC scores if they are co-activated via various signalling pathways. Although BC alone is not enough to determine which genes are regulating CS, taking BC into account with other network topological parameters can be a good indicator of gene function. Aside from high BC, CellAge genes also had a lower local clustering coefficient of 0.58, compared to a mean of 0.76 across non-CellAge genes, indicating that locally, CellAge genes connect to other genes less than the average for the network. This can also be seen at the degree level, where CellAge genes averaged only 53 connections, compared to an average of 103 connections in non-CellAge genes. Finally, the mean CC score was not significantly different between CellAge nodes and other genes in the network (0.148 in CellAge vs 0.158). CellAge genes were therefore more likely to be bottlenecks in signalling across different modules and occupy localized areas with lower network redundancy, suggesting that perturbations in their expression might have a greater impact on linking different underlying cellular processes.

The topological analysis of the main network component as a whole revealed a more modular topology than the PPI network, resulting in genes tending not to appear in multiple measures of centrality. There were 23 nodes with significant IC with senescence-related genes, including *PTPN6*, *LAPTM5*, *CORO1A*, *CCNB2* and *HPF1*. No node from the top 5 IC was present in the top 5 genes with high BC, CC, or Degree. Overall, the primary candidates of interest included *KDM4C*, which had a significant IC and was at the top 1% of CC and top 5% of BC, along with *PTPN6*, *SASH3* and *ARHGAP30*, which all had significant IC values and were at the top 5% of BC. We found that *KDM4C* and *PTPN6* have been shown to regulate CS in human cell lines, and will be added to build 2 of CellAge [65, 66].

Previous studies have advocated that measures of centrality are generally important to identify key network components, with BC being one of the most common measures. However, it has also been postulated mathematically that intra-modular BC is more important than inter-modular BC [67]. Therefore, by isolating network clusters of interest and identifying genes with high BC or centrality within submodules, we propose to identify new senescence regulators from the co-expression network.

Using the CytoCluster app (see Networks in Methods) [68], we found 54 clusters in the network, of which we represent the top clusters colored according to modularity (Module 1–16) or size (Module 17–19) (Fig. 4a). Reactome pathway enrichment for all main clusters highlighted cell cycle and immune system terms in the two largest clusters [35, 36]. The largest cluster of 460 nodes (17 CellAge nodes, Module 4), possessed a high modularity score and was strongly associated with cell cycle genes, including the following general terms: Cell Cycle; Cell Cycle, Mitotic; Mitotic Prometaphase; Resolution of Sister Chromatid Cohesion; and DNA Repair. The second largest cluster (Module 16), however, had weak modularity (ranking 26); it comprised of 450 nodes (19 CellAge nodes) and was enriched for immune-related pathways including: Adaptive Immune System; Innate Immune System; Immunoregulatory interactions between a Lymphoid and a non-Lymphoid cell; Neutrophil degranulation; and Cytokine Signaling in Immune system. Cluster 4 and Cluster 5 were not enriched for Reactome Pathways. A visual inspection showed a number of bottleneck genes between Module 1 and Module 16, consistent with the role of the immune system in clearance and surveillance of senescence cells and the secretion of immunomodulators by senescent cells [69] (Additional file 1: Table S35).

We were also interested in visualizing areas in the network with a high local clustering coefficient, as this parameter represents areas with many neighborhood interactions and, therefore, more robust areas in the network. It was found that the two clusters of interest, enriched for cell cycle terms and immune system terms, overlapped with regions of lower clustering coefficient, potentially implying parts of the biological system with less redundancy in the underlying process. Figure 4b depicts regions of high local clustering coefficient in the network (orange) and regions less well connected locally (green).

#### Unweighted microarray co-expression network

We also made an unweighted microarray co-expression network built from the COXPRESdb database of microarray gene co-expression (V6) [57] (Additional file 2: Fig. S12). Compared with the RNA-seq co-expression network, the microarray network is significantly smaller, and only included 34% of the CellAge genes (Additional file 1: Table S32). However, we found that *SMC4* was an important bottleneck in the microarray network, being in the top 5% CC and IC (Additional file 2: Fig. S12D and S12E). *SMC4* was not independently associated with senescence despite being part of the condensing II complex, which is related to cell senescence [70]. Furthermore, *SMC4* is associated with cell cycle progression and DNA repair, two key antagonist mechanisms of cell senescence development [71, 72]. *SMC4* has been linked to cell cycle progression, proliferation regulation, and DNA damage repair, in accordance to the most significantly highlighted functional clusters in the module 2 and in the whole Microarray network (Additional file 1: Table S39 and S40; Additional file 2: Fig. S13) [73, 74]. There was limited overlap between the microarray co-expression network and the RNA-seq co-expression network, although this is not surprising considering the higher specificity and sensitivity, and ability to detect low-abundance transcripts of RNA-seq [75].

#### Experimental validation of senescence candidates

**Fig. 5:**
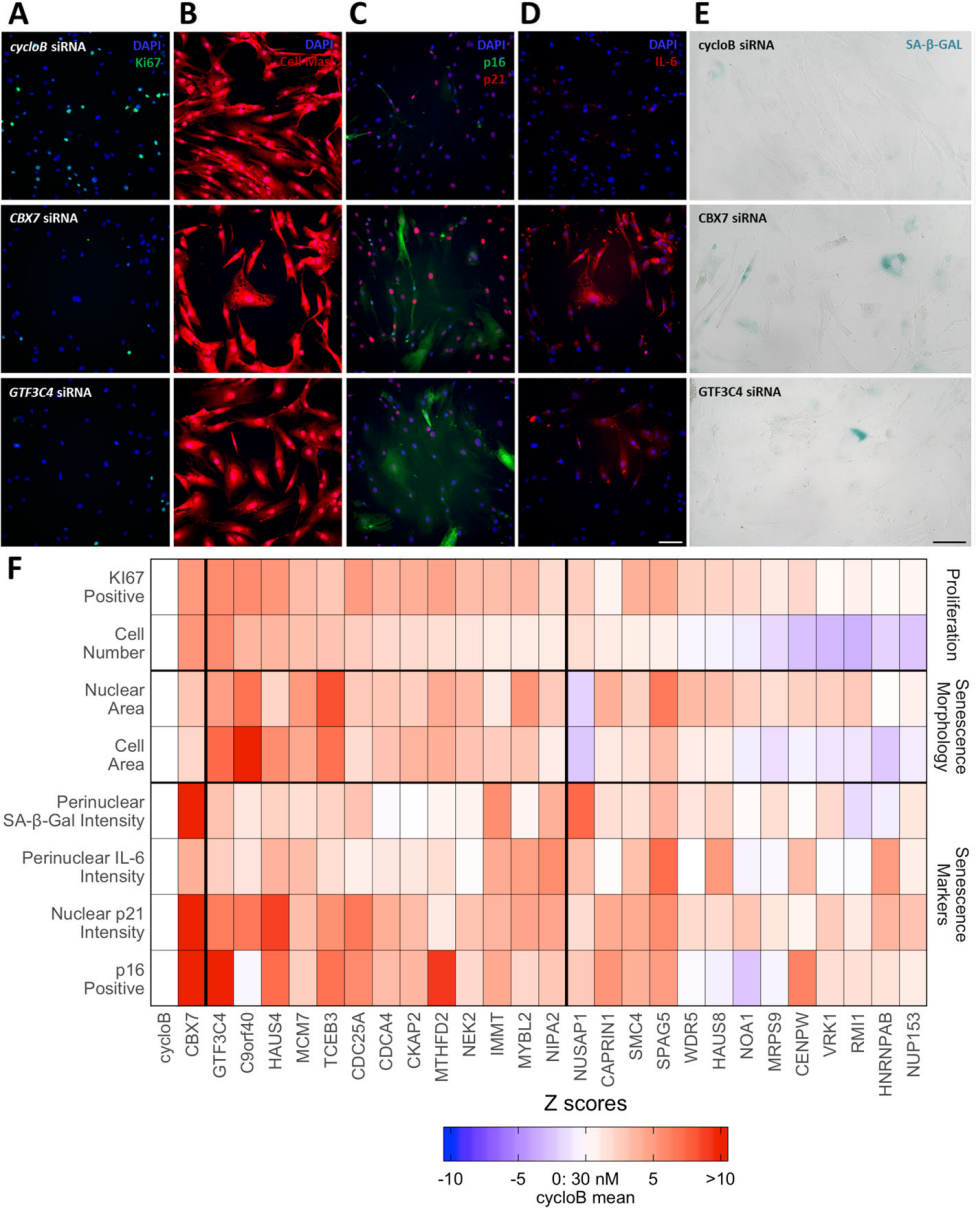
Experimental validation of 26 senescence candidates. **a–e** Representative images of fibroblasts following transfection with cyclophilin B siRNA (top row), CBX7 siRNA (middle row), or GFT3C4 siRNA (bottom row). **a** DAPI (blue) and Ki67 (green). **b** DAPI (blue) and Cell Mask (red). **c** DAPI (blue), p16 (green) and p21 (red). **d** DAPI (blue) and IL-6 (red). **e** Brightfield images following staining for SA-β-galactosidase. Size bar, 100 μm. **f** Heatmap of multiparameter analysis of proliferation markers (cell number and % Ki67 positive), senescence-associated morphology (cellular and nuclear area) and senescence markers (% p16 positive, p21 intensity, perinuclear IL-6 and perinuclear SA-β-galactosidase). Colors illustrate the number of *Z*-scores the experimental siRNA is from the cyclophilin B (cycloB) negative control mean. Data are ranked by whether or not the siRNA is a top hit (siRNAs between the thick horizontal lines), and then by the cell number *Z*-score. Red values indicate *Z*-scores that are “senescence-associated measures.” The CBX7 positive control is also shown for comparison. Data presented are from at least two independent experiments each performed with a minimum of three replicates. All *Z*-scores are available in Additional file 1: Table S44

We set out to test if candidate genes from our network analyses are indeed senescence inhibitors using a siRNA-based approach, whereby knockdowns enable the p16 and/or the p21 senescence pathway to be induced, leading to senescence [76]. We tested 26 potential senescence inhibitor candidates, 20 of which were chosen using GeneFriends, a guilt-by-association database to find co-expressed genes [59]. For this, we used the CellAge CS inhibitors as seed genes, with the assumption that genes co-expressed with senescence inhibitors would also inhibit senescence, and generated a list of the top co-expressed genes with CS inhibitors based on RNA-seq data (Additional file 1: Table S41). Furthermore, CellAge has multiple ways of partitioning genes, including the type of senescence the genes are involved in (Fig. 1b). We decided to look for genes co-expressed with stress-induced premature senescence (SIPS) inhibitors. We generated a list of genes that are co-expressed with the CellAge SIPS genes (Additional file 1: Table S42). We chose to validate five additional genes that were both co-expressed with the CellAge SIPS and are present as underexpressed in our signature of CS [32]. Finally, we chose *SMC4* from the microarray network due to its interaction with other senescence genes within the network, its association with cell cycle progression, and the fact that it is underexpressed in senescent cells, indicating it may be inhibiting senescence in replicating cells. The genes chosen, along with experimental validation results are shown in Fig. 5, while the justification for our validation and Z-scores are shown in Additional file 1: Table S43 and S44 respectively.

Next, we performed transient siRNA transfections of normal human fibroblasts using the 26 candidates and identified those siRNAs that generated the induction of a senescence phenotype, using multiparameter analysis of morphological measures and a panel of senescence markers. Senescence induction is associated with a loss of proliferation, as measured by a decrease in Ki67 index and cell number, and changes in cellular morphology, as measured by an increase in cell and nuclear area. We also quantitated changes in p16 and p21 (key senescence effectors [76]), interleukin 6 (IL-6, a common SASP marker) and SA-β-galactosidase. Knockdown of *cyclophilin B*, a housekeeper, acted as a negative control [2], while knockdown of *CBX7*, a potent senescence inhibitor, was included as a positive control for senescence induction [77]. Of the 26 genes tested, 80.7% (21/26) resulted in a decrease in Ki67 positive nuclei greater than 1 Z-score (i.e., direction of change also observed for the *CBX7* siRNA positive control, Fig. 5; Additional file 1: Table S44); 80.7% (21/26) increased p16; 96.2% increased p21 (25/26); 65.4% increase IL-6; and 65.4% (17/26) increase SA-β-galactosidase. Of the siRNAs that resulted in a decrease in Ki67 index, 61.9% (13/21) were classified as top hits as they concomitantly decreased cell number and altered at least one morphological measure. 92.3% (12/13) of the top hits activated both the p16 and p21 pathway, 84.6% (11/13) upregulated the SASP factor IL-6, while 61.5% (8/13) generated an increase in the percentage of SA-β-galactosidase positive cells. In general, we have shown the power of networks in predicting gene function, with 13 “top hits” (*GTF3C4, C9orf40, HAUS4, MCM7, TCEB3, CDC25A, CDCA4, CKAP2, MTHFD2, NEK2, IMMT, MYBL2, and NIPA2*).

## Discussion

CellAge aims to be the benchmark database of genes controlling cellular senescence and we expect it to be an important new resource for the scientific community. The development of CellAge has also provided us with the means to perform systematic analyses of CS. While showcasing the functionality of CellAge in this manuscript, we have also explored the links between CS and aging, ARDs, and cancer. At the same time, we have aimed to expand the knowledge on both the evolution and function of senescence genes, and on how CS genes interact and form genetic networks. We showed that the use of CellAge may help in identifying new senescence-related genes and we have validated several such genes experimentally. As the body of knowledge around senescence grows, it is our aim to maintain a quality resource to allow integrative analyses and guide future experiments.

We began our CellAge analysis by gaining further insight into the function of CellAge genes (Additional file 2: Fig. S3). Unsurprisingly, inducers of CS were enriched for both VEGF and TNF signalling (Additional file 1: Table S1 and S2). Secretion of VEGF is a component of the senescence phenotype and has been shown to contribute towards cancer progression [78]. Interestingly, the CellAge genes are more strongly conserved in mammals compared to other protein-coding genes, an effect not seen in worms, yeast, or flies (Additional file 1: Table S4; Additional file 2: Fig. S1A and S1B). Given the role that many of the senescence genes in CellAge play in regulating the cell cycle, it makes sense that they are evolutionarily conserved; it is not entirely surprising that there is a greater evolutionary pressure towards conserving cell cycle tumor-suppressor genes than there is towards conserving other genes. Notably, the pattern of evolutionary conservation of CS genes was found to be almost identical to that of cancer-associated genes, apparently reflecting the co-evolution between these two phenomena [53]. Nonetheless, evolutionary genomics in a comparative context allows us to have a more comprehensive understanding of the genetic bases in important phenotypic traits, like longevity [79]. During their evolutionary history, it is possible that long-lived species found ways to more efficiently solve problems related to the aging process [80, 81]. Lineages where naturally important gene regulators (e.g., TP53) have alternative molecular variants or have been lost from their genomes [82, 83] can be investigated as natural knockouts [84], since they have found a different way to solve aging-related diseases like cancer [85, 86]. We also found that the evolutionary distance between long-lived species is randomly distributed (Additional file 2: Fig. S1D; Additional file 4). Since longevity is a plastic trait that is related to multiple factors in the evolutionary history of the organisms (e.g., reproduction, body mass, habitat, metabolism, risk of predation), the way in which these genes evolved could be independent in the long-lived species analyzed.

The relationship between CS and longevity was highlighted across various sections of this manuscript. The inducers of senescence were significantly overrepresented in the anti-longevity human orthologues, while the inhibitors of senescence were even more overrepresented in the pro-longevity human orthologues (Additional file 1: Table S7) [34]. Furthermore, both the CellAge regulators of CS and the overexpressed signatures of CS were significantly overrepresented in the overexpressed aging signatures from the human, rat, and mouse aging signature meta-analysis [42]. Interestingly, we found that the overexpressed signatures of replicative CS overexpressed with age were significantly enriched for regulated exocytosis (including leukocyte activation), cell proliferation, and aging (Additional file 1: Table S10; Additional file 2: Fig. S3B). The SASP is a known inducer of chronic inflammation in aged tissue [12, 13], and the enrichment of terms relating to leukocyte activation highlights the role CS plays in activating the immune system via inflammatory factors with age. One tissue that consistently showed different CS expression patterns with age was the uterus. This observations was already noted in a previous study which also observed that DEGs downregulated in cancer were upregulated with age and DEGs upregulated in cancer were downregulated with age in six tissues, but not in the uterus [32].

CS genes are not expressed in a tissue-specific manner (Additional file 1: Table S11; Additional file 2: Fig. S4) and less than half of the CS genes undergo a significant change in expression with age (Fig. 2; Additional file 2: Fig. S5A), suggesting that the pathways triggering differential expression of CS genes with age are shared between cells across tissues. Indeed, we found that *CDKN2A* was overexpressed in 19 human tissues with age, albeit only significantly so in 10 of the tissues (Additional file 1: Table S18) [32]. Nonetheless, across all simulations, CS genes significantly overexpressed across multiple tissues with age by chance never exceeded seven tissues (Fig. 2d; Additional file 1: Table S17 and S19). The significant increase in *CDKN2A* expression across a significant number of human tissues with age is an indicator that at least some cell types are undergoing CS with age. *ZMAT3*, an inhibitor of CS, was also significantly overexpressed with age in seven tissues, including blood vessel, lung, and prostate, which also had significant increases in *CDKN2A* expression. Indeed, both *ZMAT3* and *CDKN2A* were overexpressed across the majority of GTEx tissues with age (Additional file 2: Fig. S5D). Furthermore, ~ 40% of the CellAge database was compiled using experiments exclusively in human fibroblast cell lines. Of the 20 studies used to compile the signatures of CS, 10 also performed gene manipulation experiments on fibroblasts [32]. Fibroblasts are present in connective tissues found between other tissue types across the human body, and the tissue samples analyzed to compile GTEx likely contained fibroblast gene expression. This may partially explain the lack of tissue-specific CellAge genes. It is further unclear whether the trends in differential expression of the CellAge genes we see across aged human tissue samples is a result of fibroblast senescence, or if heterogenous gene populations are undergoing CS. We have partially addressed this issue by doing subgroup analysis of CellAge genes confirmed to control senescence outside of fibroblast cell lines and found that the overlap between these genes and both the signatures of aging and cancer genes is still significant.

We found a strong association between senescence and neoplastic diseases (Additional file 1: Table S21). This is not surprising given the known role of senescence in tumor suppression. Some CS genes were also shared between many of the ARD classes. These results are in line with a previous analysis investigating the relationship between CS and ARD genes carried out using different datasets [53]. Tacutu et al reported significant overlaps (i.e., 138 genes – 53% – in common between CS and cancer vs 21–8% – between CellAge and neoplasms); many more than we did. The study found that many genes shared between CS and several non-cancer ARDs are also involved in cancer. While removing cancer genes from our ARD dataset did not result in such a striking effect, it nonetheless substantially cut the number of overlaps to a statistically insignificant level, adding weight to the hypothesis that cancer genes have a bridging role between CS and ARDs. Furthermore, we found a significant overlap between both the CellAge inhibitors and inducers of senescence, and oncogenes and TSG (Fig. 3). Genes that induce senescence, however, tended to be tumor suppressors, while genes that inhibit senescence tended to be oncogenes, a finding that is consistent with the classical view of cellular senescence as a tumor-suppressor mechanism.

We next explored what information could be obtained by applying a network analysis to CellAge. From the list of CellAge genes, three networks of CS were generated: a PPI network and two co-expression networks, with the aim of identifying new senescence regulators based primarily on network centrality of the genes.

The examination of the PPI network to identify possible regulators based on centrality revealed 25 central genes in the network, ranking in the top 1% in at least two network topological parameters (degree, BC, CC, or IC) (Additional file 1: Table S33). However, 13 of these genes are already in the CellAge database, and we found 11 of these genes have already been shown to drive CS in human cell lines and will be added into build 2 of CellAge.

We looked at the RNA-Seq co-expression network in detail, using the main connected component of 3198 genes to find highly central genes to the network as a whole, and those occupying subnetworks of interest. The RNA-Seq was a highly modular network, separated into some subnetworks of distinct functions (Fig. 4). The two largest and more central networks contained a number of known senescence genes. We expanded the analysis of these networks in particular, identifying a number of bottleneck nodes. Cluster 1 was enriched for cell cycle processes, which is not overly surprising given that senescence involves changes in cell cycle progression. However, cluster 2 comprised of enriched terms relating to immune system function. One of the aims in biogerontology is to understand and reverse the effects of aging on the immune system. Additional file 1: Table S38 highlights the genes in both clusters that are potential CS bottlenecks within the network and may warrant further study.

Using siRNAs, we were able to test the potential role of 26 gene candidates in inhibiting senescence (Fig. 5). The list of candidates was primarily compiled using CellAge inhibitors as seeds to generate co-expressed genes in GeneFriends, a collection of RNA-seq co-expression data [59] (Additional file 1: Table S43). Of the 26 genes, 13 were top hits, decreasing cell number, altering at least one morphological measure, and activating the p16 and/or p21 pathway. Additional file 1: Table S45 highlights the four CS candidates we found that have not yet been associated with senescence. We have showcased how co-expression networks can be used to accurately infer senescence gene candidates, which can then be experimentally verified.

## Conclusion

Overall, our CellAge database is the first comprehensive cellular senescence database, which will be a major resource for researchers to understand the role of senescence in aging and disease. Besides, we found that CS genes are conserved in vertebrates but not invertebrates and that genes related to the CS tend not to be tissue-specific. We observed that genes inducing CS trended towards upregulation with age across most human tissues, and these genes are overrepresented in both anti-longevity and tumor-suppressing gene datasets, while genes inhibiting senescence were not overexpressed with age and were overrepresented in pro-longevity and oncogene datasets. CS genes were also overrepresented in genes linked to aging-related diseases, primarily in neoplasms.

Using network biology, we implicated the CellAge genes in various processes, particularly cell division and immune system processes. We used network topology to identify potential regulators of CS and bottlenecks that could impact various downstream processes if deregulated. Indeed, we identified 11 genes that have already been shown to contribute towards CS, which will be added to future versions of CellAge. Finally, we experimentally verified 26 genes that induce CS morphology or biomarkers when knocked down in human mammary fibroblasts. Of these, 13 genes (*C9orf40*, *CDC25A*, *CDCA4*, *CKAP2*, *GTF3C4*, *HAUS4*, *IMMT*, *MCM7*, *MTHFD2*, *MYBL2*, *NEK2*, *NIPA2*, and *TCEB3*) were strong hits in inducing a senescent phenotype.

Cellular senescence is one of the hallmarks of aging [87] and the accumulation of senescent cells in human tissues with age has been implicated as a driver of aging-related diseases. Indeed, pharmacological approaches targeting senescent cells, like senolytics, are a major and timely area of research that could result in human clinical applications [5, 88]. It is imperative that we fully understand and deconstruct cellular senescence in order to target aging-related diseases. We hope that CellAge will help researchers understand the role that CS plays in aging and aging-related diseases and contributes to the development of drugs and strategies to ameliorate the detrimental effects of senescent cells.

## Methods

### CellAge compilation

CellAge was compiled following a scientific literature search, manual curation, and annotation, with genes being appended to the database if they met the following criteria:

- Only gene manipulation experiments (gene knockout, gene knockdown, partial or full loss-of-function mutations, overexpression or drug-modulation) were used to identify the role of the genes in cellular senescence. The search focussed on genes from genetic manipulation experiments to ensure objectivity in the selection process.
- The genetic manipulation caused cells to induce or inhibit the CS process in the lab. Cellular senescence was detected by growth arrest, increased SA-β-galactosidase activity, SA-heterochromatin foci, a decrease in BrdU incorporation, changes in morphology, and/or specific gene expression signatures.
- The experiments were performed in primary, immortalized, or cancer human cell lines.

40% of the experiments were conducted exclusively in fibroblasts. The data was compiled from 230 references. The curated database comprises cell senescence genes together with a number of additional annotations useful in understanding the context of each identified CS gene (Additional file 1: Table S46).

We categorized genes according to three types of senescence: replicative, oncogene-induced or stress-induced. Replicative senescence was the default category, while genes were listed as oncogene-induced if the reference explicitly mentioned the gene induced or delayed oncogene-induced senescence. Finally, stress-induced senescence was used to indicate that the gene was necessary to induce or inhibit senescence caused by external stressors like drugs/chemicals, serum deprivation, or radiation. We also recorded whether a gene induces or inhibits CS. For example, a gene whose overexpression is associated with increased senescence is classified with the “induces” tag, whereas if the overexpression of a gene inhibits senescence, then it is classified with the “inhibits” tag. Similarly, if the knockout or knockdown of a gene induces senescence, then it is recorded with the “inhibits” tag. Together with the annotations identified in Additional file 1: Table S46, we also incorporated a number of secondary annotations into the database such as various gene identifiers, the gene description, gene interaction(s), and quick links to each senescence gene. The CellAge database also provides crosslinks to genes in other HAGR resources, i.e., GenAge, GenDR and LongevityMap, which we hope will enable inferences to be made regarding the link between human aging and CS.

### CellAge data sources

Build 1 of CellAge resulted in a total of 279 curated cell senescence genes which we have incorporated into the HAGR suite of aging resources. The HAGR platform comprises a suite of aging databases and analysis scripts. The CellAge interface has been designed with the help of JavaScript libraries to enable more efficient retrieval and combinatorial searches of genes. As with the other HAGR databases, we have used PHP to serve the data via an Apache web server. The raw data can be downloaded via the main HAGR downloads page in CSV format or filtered and downloaded from the main search page.

The first part of our work consisted in finding which genes driving CS are also associated with ARDs or with longevity, using the following data sources:

- Human genes associated with CS: CellAge build 1.
- Human genes associated with human aging: GenAge human build 19.
- Human orthologues of model organisms’ genes associated with longevity: proOrthologuesPub.tsv and antiOrthologuesPub.tsv file (https://github.com/maglab/genage-analysis/blob/master/Dataset_4_aging_genes.zip) [34].
- Human oncogenes: Oncogene database (http://ongene.bioinfo-minzhao.org/index.html).
- Human tumor suppressor gene database: TSGene 2.0 (https://bioinfo.uth.edu/TSGene/index.html).
- Human genes associated with ARDs (https://github.com/maglab/genage-analysis/blob/master/Dataset_5_disease_genes.zip) [34]. This data concerns the 21 diseases with the highest number of gene associations, plus asthma, a non-aging-related respiratory system disease used as a control.
- Human genes differentially expressed with age from the GTEx project (v7, January 2015 release) [32, 43].

### CellAge data analysis

Statistical significance was determined by comparing the *p*-value of overlapping CellAge gene symbols with the different data sources, computed via a hypergeometric distribution and Fisher’s exact test. We used PubMed to understand the relative research focus across the protein-coding genome and incorporate this into the analysis to account for publication bias. We used BioMart to obtain approximately 19,310 protein-coding genes, then using an R script we queried NCBI for the publication results based on the gene symbol using the following query [89, 90]:

(“***GENE_SYMBOL***”[Title/Abstract] AND Homo [ORGN]) NOT Review [PTYP]

The GENE_SYMBOL was replaced in the above query by each of the genes in turn. Certain genes were removed as they matched common words and, therefore, skewed the results: *SET*, *SHE*, *PIP*, *KIT*, *CAMP*, *NODAL*, *GC*, *SDS*, *CA2*, *COPE*, *TH*, *CS*, *TG*, *ACE*, *CAD*, *REST*, *HR*, and *MET*. The result was a dataframe in R comprising variables for the “gene” and the “hits.” We used the R package called “rentrez” to query PubMed for the result count [91].

### Evolution of CellAge genes

The percentage of CellAge genes with orthologues in *Rhesus macaque, Rattus norvegicus, Mus musculus, Saccharomyces cerevisiae, Caenorhabditis elegans*, and *Drosophila melanogaster* were found using Biomart version 88 by filtering for genes with “one2one” homology and an orthology confidence score of one [89]. We also found the total number of human genes with orthologues in the above species using Biomart. Significance was assessed using a two-tailed z-test with BH correction.

The phylogenetic arrangement included twenty-four species representative of major mammalian groups. The genomes were downloaded in CDS FASTA format from Ensembl (http://www.ensembl.org/) and NCBI (https://www.ncbi.nlm.nih.gov/) (Additional file 1: Table S6).

To remove low quality sequences we used the clustering algorithm of CD-HITest version 4.6 [92] with a sequence identity threshold of 90% and an alignment coverage control of 80%. The longest transcript per gene was kept using TransDecoder.LongOrfs and TransDecoder. Predict (https://transdecoder.github.io) with default criteria [93]. In order to identify the orthologs of the 279 CellAge human genes in the other 23 mammalian species, the orthology identification analysis was done using OMA standalone software v. 2.3.1 [41]. This analysis makes strict pairwise sequence comparisons “all-against-all,” minimizing the error in orthology assignment. The orthologous pairs (homologous genes related by speciation events) are clustered into OrthoGroups (OG) [94]; this was done at the Centre for Genomic Research computing cluster (Linux-based) at the University of Liverpool. The time calibrated tree was obtained from TimeTree (http://www.timetree.org/) and the images were downloaded from PhyloPic (http://phylopic.org/).

In order to structure the evolutionary distance for the CellAge genes between the five long-lived mammals and the others 19 mammalian species, the amino acid sequences from the 271 CellAge OrthoGroups were aligned using the L-INS-i algorithm from MAFFT v.7 [95]. Ambiguous and missing sites were removed from the alignments using the pxclsq function from phyx [96]. We concatenated the amino acid alignments using the concat function from AMAS [97] for the 271 CellAge genes. To analyze the variation of the CellAge genes in mammals, we obtained the branch lengths using log-likelihood for a fixed tree through IQ-TREE [98] for (a) the concatenated alignment (271 CellAge genes) and (b) the 22 CellAge genes conserved among the 24 mammalian species in order to understand the individual gene evolution. The topology of reference was the phylogenetic tree from TimeTree.

We used the Faith’s phylogenetic diversity index (PD) [99] through the “picante” R package [100] to calculate the evolutionary distances. The Faith’s PD index was used to calculate the sum of the total phylogenetic branch length for one or multiples species. We calculated the observed Faith’s PD from our data and we compared the results with the expected Faith’s PD (expected.pd) using a binomial sampling with a fixed probability of each tip being sampled.

### Overlap analysis

We conducted overlap analysis using R to understand how the CellAge genes and signatures of CS were differentially expressed with GenAge, ARD, and cancer genes. We also examined the overlap between CS genes and differentially expressed signatures of aging [42], and genes differentially expressed in various human tissues with age. Fisher’s exact test was used on the contingency tables and significance was assessed by *p* values adjusted via Benjamini-Hochberg (BH) correction. For the comparison of genes differentially expressed in at least one tissue with age between the CS genes and the genome, some genes were differentially expressed in opposite directions across numerous tissues (Additional file 2: Fig. S5A). Genes differentially expressed in both directions were added to the overexpressed and underexpressed DEGs in each CS gene list, and to the total number of genes in the genome to compensate for the duplicate gene count (Additional file 1: Table S14 and S15). Fisher’s exact test was also used to test for significance of tissue-specific CellAge gene expression. Significance of overlap analysis between CellAge and LAGs was computed using a hypergeometric distribution and FDR was corrected using Bonferroni correction. The GeneOverlap package in R was used to test for overlaps between the CellAge inducers and inhibitors of senescence, and the oncogenes and TSGs [101]. Results for all overlap analyses were plotted using the ggplot2 library [90, 102].

### Simulation of CS gene expression in human aging

The RNA-seq gene expression data on GTEx was scrambled in such a way that all protein-coding genes in each tissue were assigned a random paired p and log_2_FC value from the original gene expression data of each respective tissue. The randomly sorted gene expression data was then filtered for significance (*p* < 0.05, moderated *t*-test with BH correction, absolute log_2_FC > log_2_(1.5)) [32, 103], and the CellAge accessions were extracted and overlapped across all the simulated expression data in 26 tissues from GTEx. The probability of a CS gene being overexpressed or underexpressed across multiple tissues by chance was calculated across 10,000 simulations.

### Functional enrichment

The analysis of CellAge included gene functional enrichment of the database. We used DAVID functional clustering (https://david.ncifcrf.gov/) to identify functional categories associated with CellAge [35, 36].

The Overrepresentation Enrichment Analysis (ORA) of biological processes (Gene Ontology database) was done via the WEB-based Gene SeT AnaLysis Toolkit (WebGestalt) for the analysis of all CellAge genes, CellAge CS regulators and overexpressed signatures of CS overexpressed in the meta-analysis of aging signatures, and for the CellAge genes overlapping with tumor suppressor and oncogenes [38]. A *p* value cutoff of 0.05 was used, and *p* values were adjusted using BH correction. Redundant GO terms were removed and the remaining GO terms were grouped into categories based on their function using the default parameters on Reduce + Visualize Gene Ontology (REVIGO) [37]. Results were then visualized using and the R package treemap [104] (Fig. 1c; Additional file 2: Fig. S8A – S8D). Venn diagrams to represent gene overlaps were created using Venny [52] and the ggplot2 library [90, 102].

### Networks

We used Cytoscape version 3.6.1 to generate networks and R version 3.3.1 to perform aspects of the statistical analysis [90, 105]. The networks were built starting from a list of seed nodes—all genes included in build 1 of CellAge, part of the Human Ageing Genomic Resources [28]. Network propagation was measured using the Cytoscape plugin Diffusion [106].

The analysis of the fit to the scale-free structure was calculated by the Network Analyzer tool of Cytoscape 3.2.1 [105]. Network analyzer is a Cytoscape plugin which performs topological analysis on the network and reports the pillar nodes on the network structure based on a series of mathematical parameters. Network analyzer also calculates the fit of the distribution of the number of edges per node to the power law distribution. A significant fit to the power law indicates the presence of a scale-free structure in the network [61, 107]. The analysis was applied to the PPI network, the RNA-seq Unweighted Co-expression network, and the Microarray Unweighted Co-expression network of cellular senescence (Additional file 2: Fig. S9). The Network Analyzer tool was also used to calculate BC, CC, and IC in the networks.

#### Protein-protein interaction network

The protein-protein interaction network was built from the BioGrid database of physical multi-validated protein interactions (Biology General Repository for Interaction Datasets) version 3.4.160, using CellAge proteins as seed nodes and extracting the proteins encoded by CellAge genes as well as the first-order interactors of CellAge proteins [108]. After removing duplicated edges and self-loops, the network consisted of 2643 nodes and 16,930 edges. The network was constructed and visualized in Cytoscape version 3.6.1. The “CytoCluster” App in Cytoscape was used to identify modules in the network with the following parameters: HC-PIN algorithm; Weak, Threshold = 2.0; ComplexSize Threshold = 1% [68].

#### Unweighted RNA-Seq co-expression network

The RNA-seq co-expression network was built using CellAge data and RNA-Seq co-expression data taken from Genefriends (http://genefriends.org/RNAseq) [59].

The unweighted co-expression network was built applying the method of correlation threshold selection described by Aoki to the GeneFriends database of RNA-Seq co-expression version 3.1 [109]. Aoki initially designed this methodology for plant co-expression network analysis, but it has been successfully applied to build human networks [110]. The Pearson Correlation Coefficient (PCC) threshold which generated the database of edges with the lowest network density was selected. The network density is the proportion of existing edges out of all possible edges between all nodes. The lower the network density is the more nodes and fewer edges are included in the network. The lower the number of edges, the higher the minimum correlation in expression between each pair of genes represented by the edges. The higher the number of nodes, the higher the portion of nodes from CellAge included, and, therefore, the more representative the network is of the CellAge database. The PCC threshold of 0.65 generated the database of interactions of RNA-Seq co-expression with the lowest network density, 0.01482 (Additional file 2: Fig. S14A). The unweighted RNA-Seq network was generated and visualized in Cytoscape 3.6.1.

#### Microarray co-expression network

The microarray co-expression network was generated using the CellAge genes as seed nodes and their direct interactions and edges, derived using the COXPRESdb database of Microarray co-expression (version Hsa-m2.c2-0) [57]. PCC threshold of 0.53 created the Microarray database with the lowest network density, 1.006 × 10^− 2^ (Additional file 2: Fig. S14B). The adjustment of the node-degree distribution to the power law distribution had a correlation of 0.900 and an R-squared of 0.456 (Additional file 2: Fig. S9C). The fit to the power law distribution confirmed the scale-free structure of the network.

### Experimental validation of new CS genes

We used normal human mammary fibroblasts (HMFs) and siRNAs to find new CS regulators based on high-ranking co-expressed inhibitors of CS and SIPS inhibitors. We also tested *SMC4* due to its high-scoring topological parameters within the microarray co-expression network (see Experimental Validation of Senescence Candidates in Results).

#### Cell culture and reagents

Fibroblasts were obtained from reduction mammoplasty tissue of a 16-year-old individual, donor 48 [111]. The cells were seeded at 7500 cells/cm^2^ and maintained in Dulbecco’s modified Eagle’s medium (DMEM) (Life Technologies, UK) supplemented with 10% fetal bovine serum (FBS) (Labtech.com, UK), 2 mM l-glutamine (Life Technologies, UK) and 10 μg/mL insulin from bovine pancreas (Sigma). All cells were maintained at 37 °C/5% CO_2_. All cells were routinely tested for mycoplasma and shown to be negative.

#### siRNA knockdown experiments

For high-content analysis (HCA), cells were forward transfected with 30 nM siRNA pools at a 1:1:1 ratio (Ambion) using Dharmafect 1 (Dharmacon) in 384-well format. Control siRNA targeting cyclophilin B (Dharmacon) or Chromobox homolog 7 (CBX7, Ambion) were also included as indicated. Cells were incubated at 37 °C/5% CO_2_ and medium changed after 24 h. Cells were then fixed/stained 96 h later and imaged as described below. The siRNA sequences are provided in Additional file 1: Table S47A and S47B.

#### *Z*-score generation

For each of the parameters analyzed, significance was defined as one *Z*-score from the negative control mean and average *Z*-scores from at least two independent experiments performed in at least triplicate are presented. *Z*-scores were initially generated on a per experiment basis according to the formula below:

$$ Z-\mathrm{score}=\left(\mathrm{mean}\ \mathrm{value}\ \mathrm{of}\ \mathrm{target}\ \mathrm{siRNA}-\mathrm{mean}\ \mathrm{value}\ \mathrm{for}\ \mathrm{cyclophilin}\ \mathrm{B}\ \mathrm{siRNA}\right)/\mathrm{standard}\ \mathrm{deviation}\ \left(\mathrm{SD}\right)\ \mathrm{for}\ \mathrm{cyclophilin}\ \mathrm{B}\ \mathrm{siRNA}. $$

#### Immunofluorescence microscopy and high-content analysis

Cells were fixed with 3.7% paraformaldehyde, permeabilized for 15 min using 0.1% Triton X and blocked in 0.25% BSA before primary antibody incubations. Primary antibodies used are listed in Additional file 1: Table S48. Cells were incubated for 2 h at room temperature with the appropriate AlexaFluor-488 or AlexaFluor-546 conjugated antibody (1:500, Invitrogen), DAPI, and CellMask Deep Red (Invitrogen). Images were acquired using the IN Cell 2200 automated microscope (GE), and HCA was performed using the IN Cell Developer software (GE).

## Supplementary information

Additional file 1: Supplementary Tables. Excel file containing Supplementary Tables S1-S48.

Additional file 2: Supplementary Figures. PDF containing Supplementary Fig. S1-S14.

Additional file 3. 271 CellAge orthogroups. Directory containing FASTA files of CellAge orthogroups.

Additional file 4. Evolutionary distance in CellAge genes. PDF with Faith’s phylogenetic diversity index of 22 individual CellAge genes conserved amongst all 24 mammalian species.

Additional file 5. GTEx simulated expression script. R script to find expected number of overlaps between GTEx tissue DEGs with age when gene names are scrambled.

Additional file 6. Review history.

## Data Availability

Supplementary figures and citations, tables, and files are available on the Integrative Genomics of Ageing Group CellAge_supplementary GitHub repository, along with an R script to recreate the scrambled GTEx gene expression data (Additional file 5) (https://github.com/maglab/CellAge_supplementary) [112]. CellAge, GenAge, and disease genes are available on HAGR (https://genomics.senescence.info/) [28, 34]. Tissue-specific differentially expressed genes with age and signatures of cellular senescence are from [32].
