# Supplementary material for: A multidimensional systems biology analysis of cellular senescence in aging and disease
Source: Genome Biol. 2020 Apr 7;21:91. doi: 10.1186/s13059-020-01990-9 (PMC7333371; doi:10.1186/s13059-020-01990-9)
Supplement: Supplementary file 2 — PDF containing Supplementary Fig. S1-S14. [file 13059_2020_1990_MOESM2_ESM.pdf]

Fig. S1

A

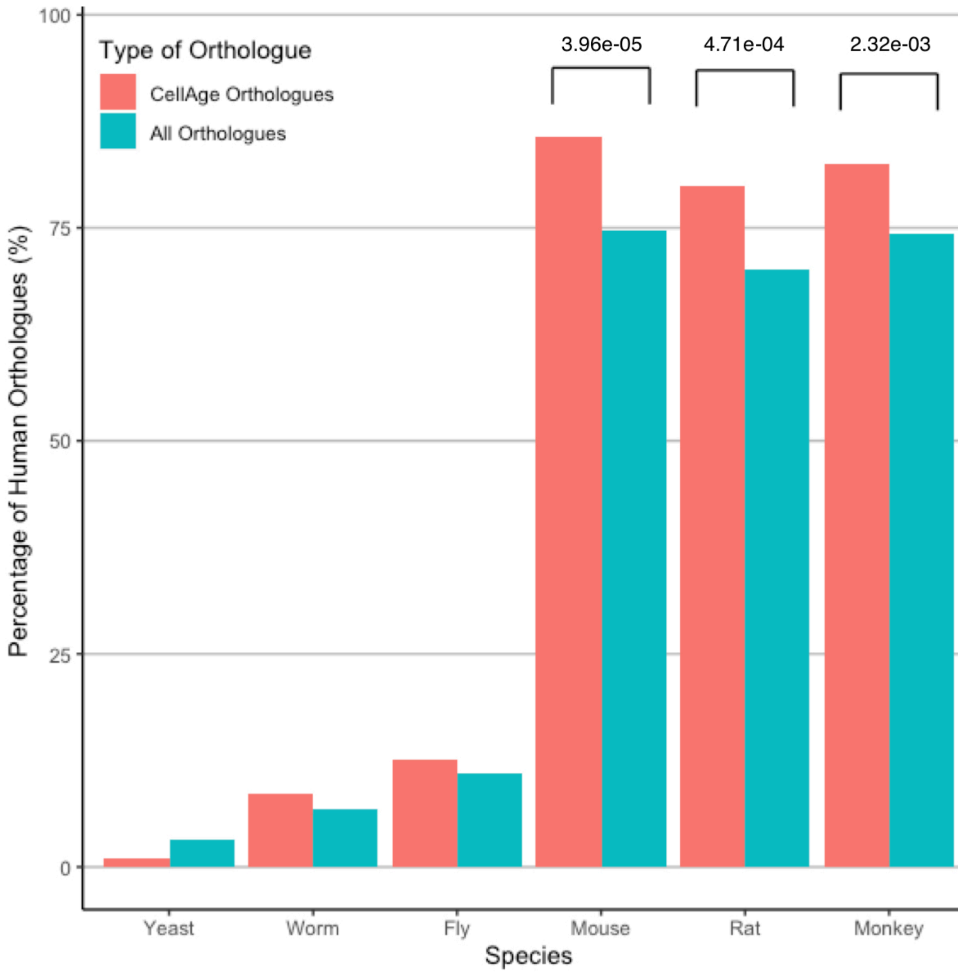

Type of Orthologue
CellAge Orthologues
All Orthologues
3.96e-05
4.71e-04
2.32e-03
100
75
50
25
0
Percentage of Human Orthologues (%)
Yeast
Worm
Fly
Mouse
Rat
Monkey
Species


B

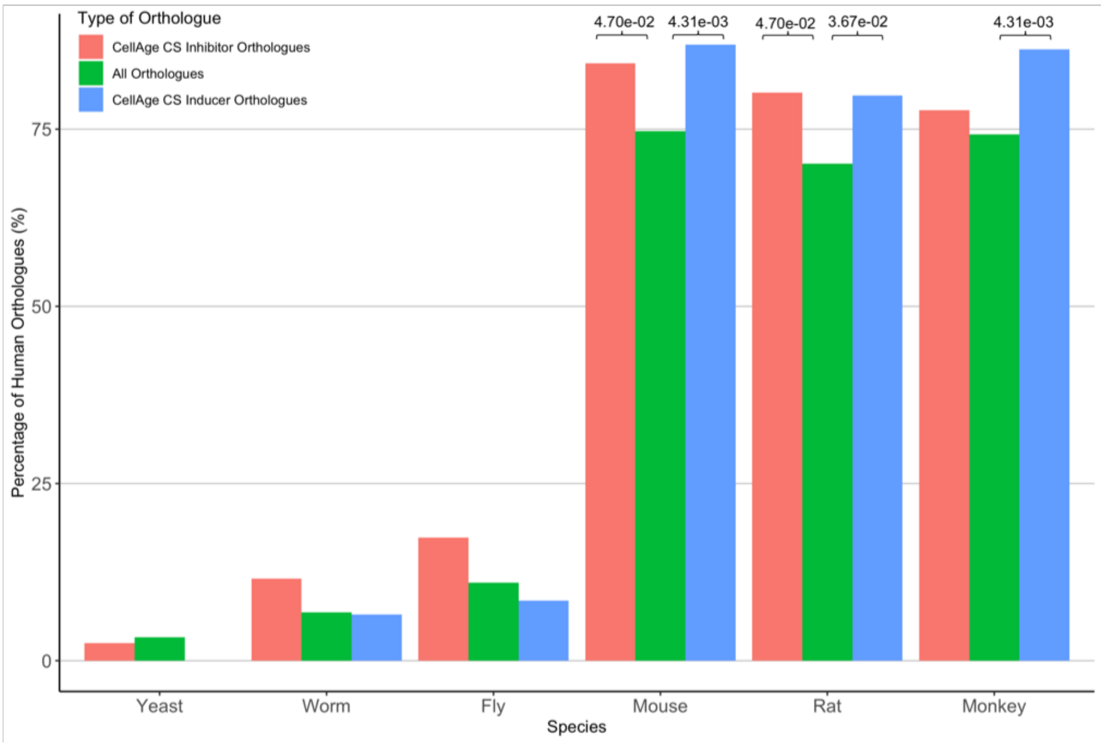

Type of Orthologue
CellAge CS Inhibitor Orthologues
All Orthologues
CellAge CS Inducer Orthologues
4.70e-02
4.31e-03
4.70e-02
3.67e-02
4.31e-03
75
50
25
0
Percentage of Human Orthologues (%)
Yeast
Worm
Fly
Mouse
Rat
Monkey
Species

Fig. S1

C

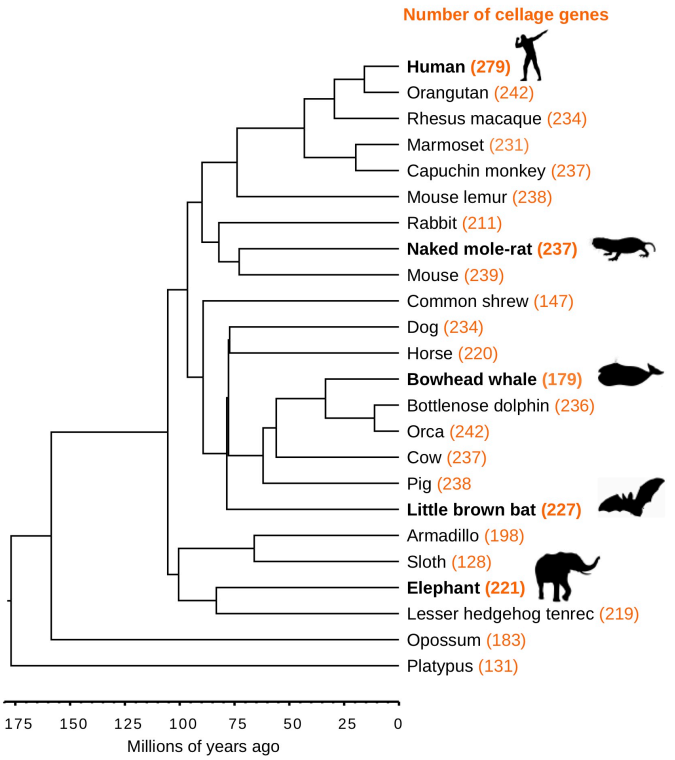


D

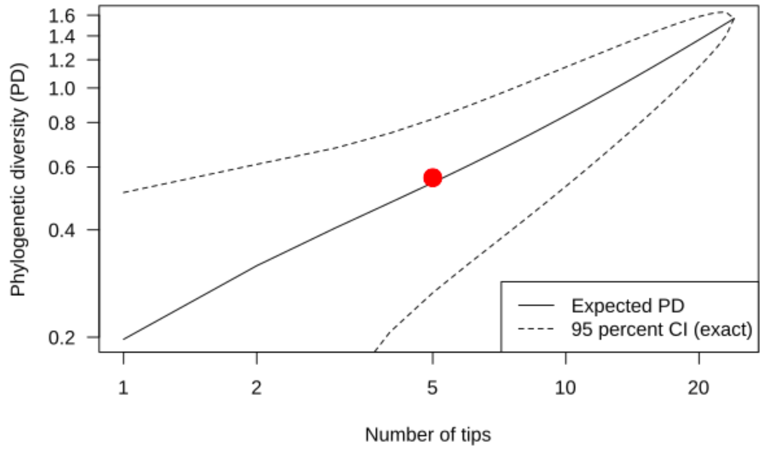


**Fig. S1. Evolution of the CellAge genes (A) Comparing conservation of the CellAge genes and normal genes in human vs model organisms. * Indicates significant differences between conservation ($p<0.05$, z-test with BH correction). (B) Comparing conservation of the CellAge inducers and inhibitors of CS and normal genes in human vs model organisms. * Indicates significant differences between conservation ($p<0.05$, z-test with BH correction). (C) Orthologous CellAge genes present in 24 mammalian species. The number of orthologues are shown in orange. The species in bold are considered long-lived with medical importance in anti-ageing research. (D) Faith's phylogenetic diversity index (PD) with a concatenated tree (271 CellAge genes) for 24**

Fig. S1

**mammals, including five long-lived species. The red dot indicates the Faith's PD index observed from the data.**

Fig. S2

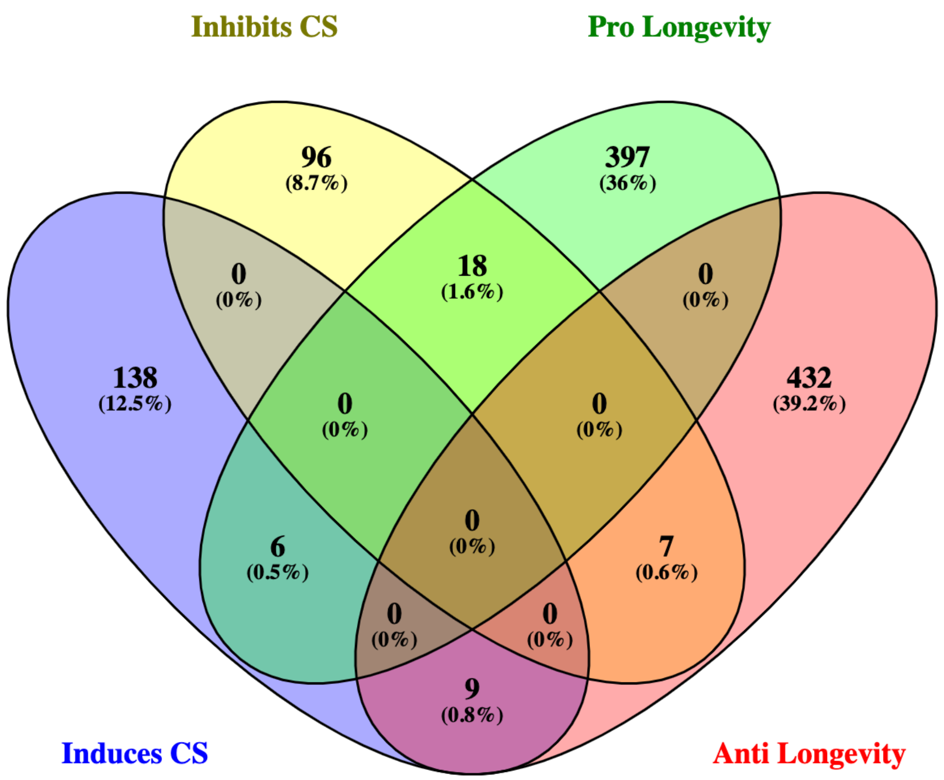


**Fig. S2. Overlap between CellAge inducers and inhibitors, and pro and anti-longevity genes.**

Fig. S3

**A**

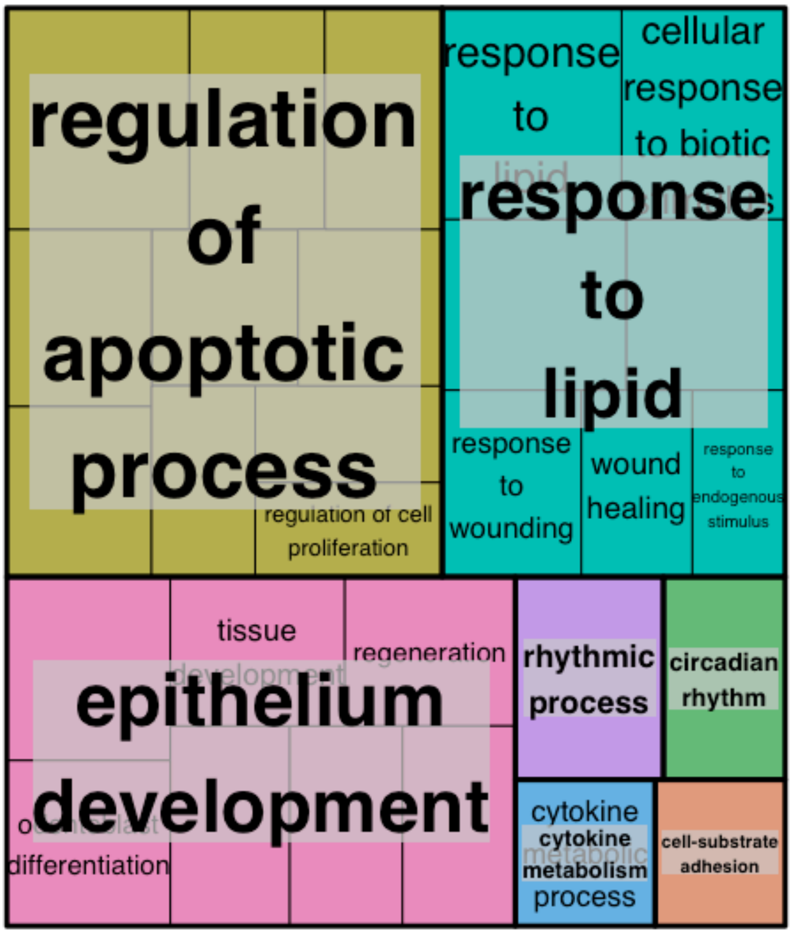


**B**

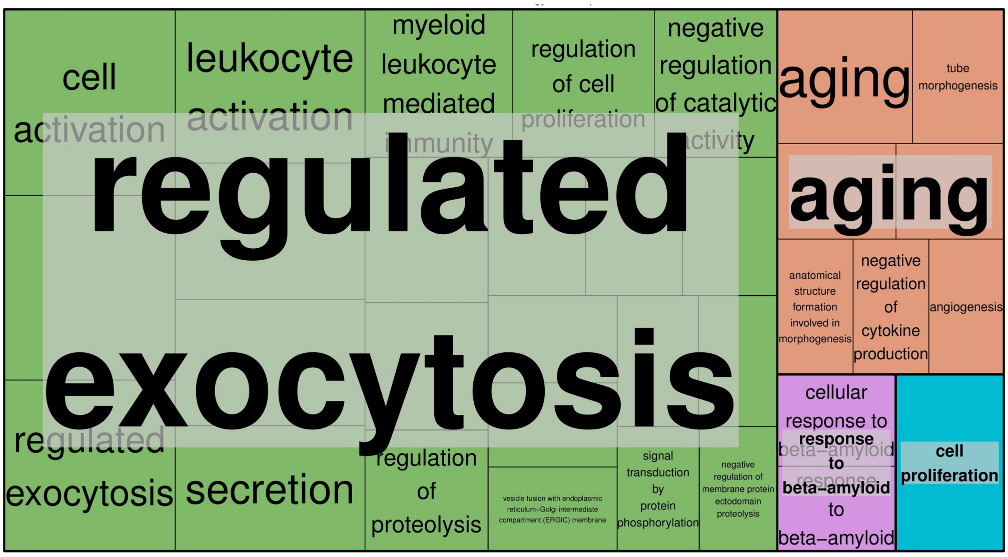


**Fig. S3. Treemaps of enriched GO terms for (A) CellAge inducers of CS overexpressed with age and (B) overexpressed signatures of CS overexpressed with age.**

Fig. S4

**A**

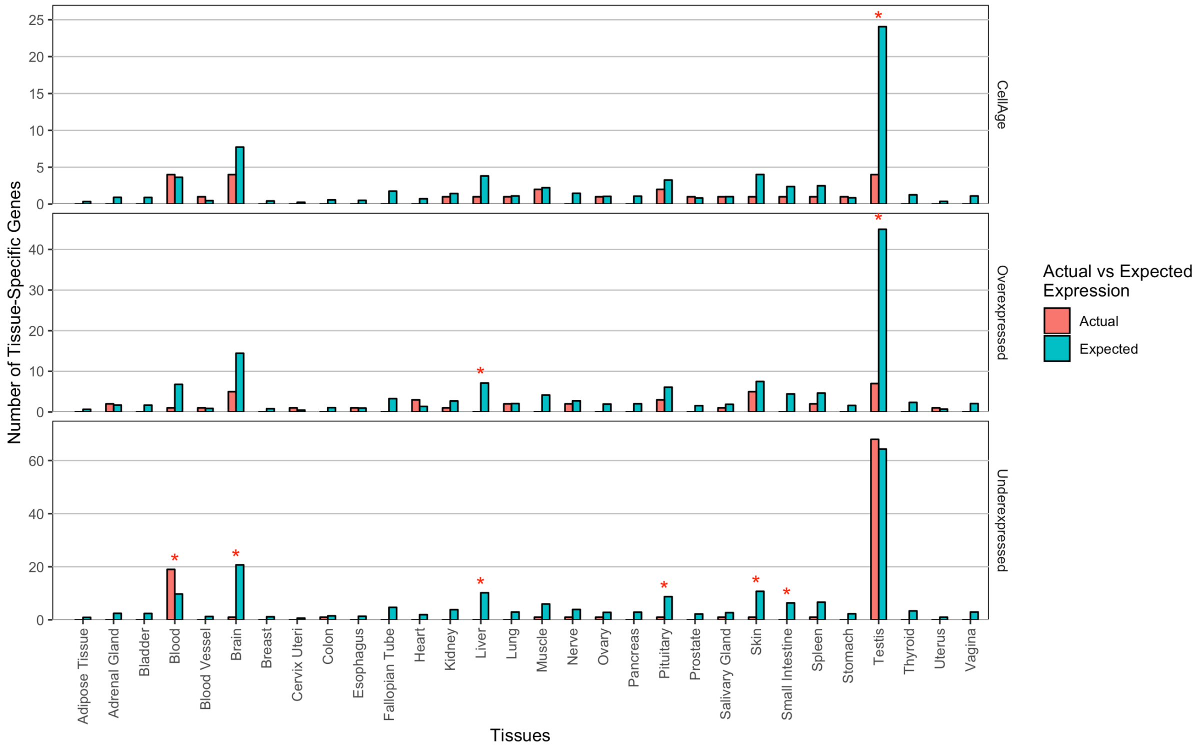


**B**

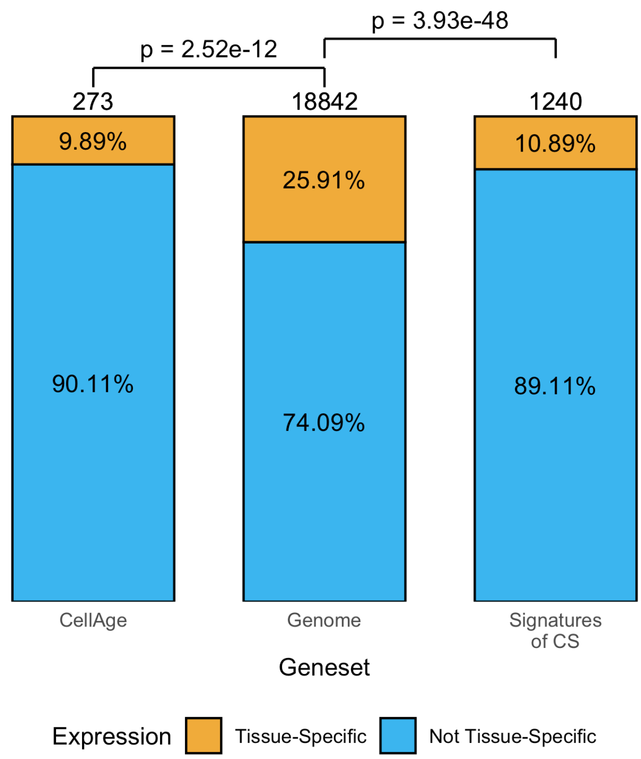


**Fig. S4. (A) Comparison between the actual number of CS genes expressed in a tissue-specific manner vs. the expected number. * Represents a significant difference (p<0.05, Fisher's exact test**

Fig. S4

**with BH correction). Full data available in SI Table 9. (B) Comparison between the ratio of tissue-specific to non-tissue-specific genes in the CS datasets vs. all protein-coding genes. P-values denote significance ($p<0.05$, Fisher's exact test with BH correction).**

Fig. S5

**A**

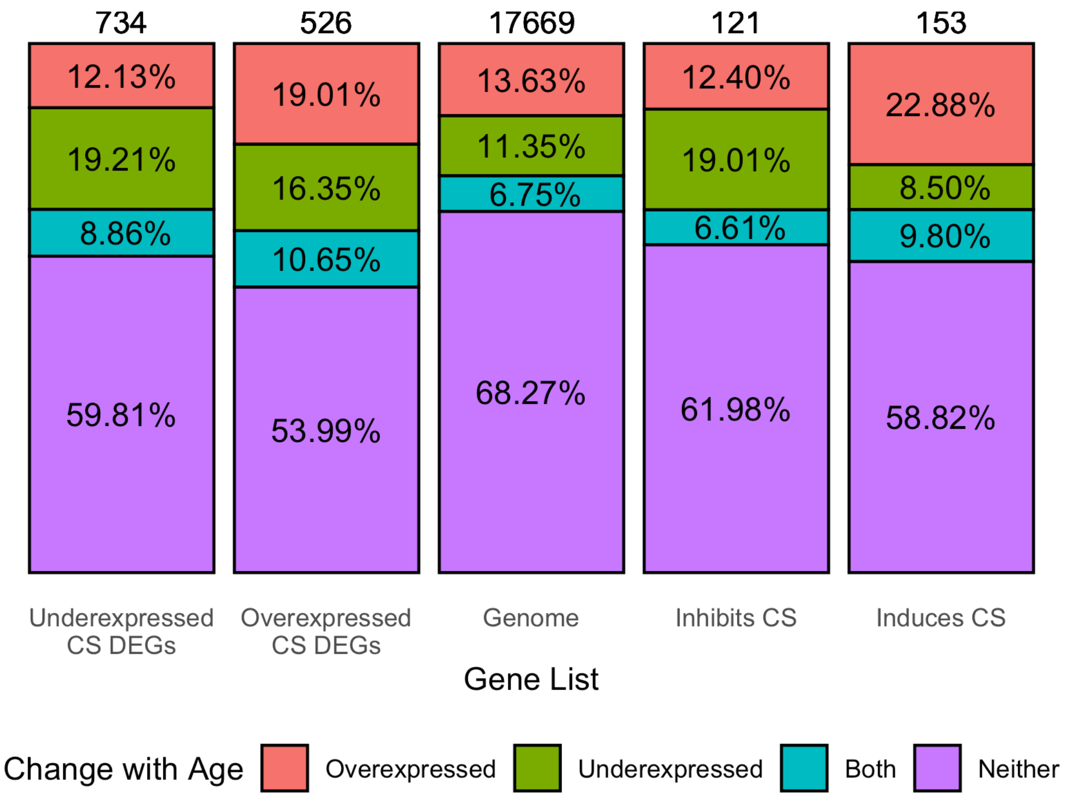

734
526
17669
121
153
12.13%
19.21%
8.86%
59.81%
19.01%
16.35%
10.65%
53.99%
13.63%
11.35%
6.75%
68.27%
12.40%
19.01%
6.61%
61.98%
22.88%
8.50%
9.80%
58.82%
Underexpressed CS DEGs
Overexpressed CS DEGs
Genome
Inhibits CS
Induces CS
Gene List
Change with Age
Overexpressed
Underexpressed
Both
Neither


**B**

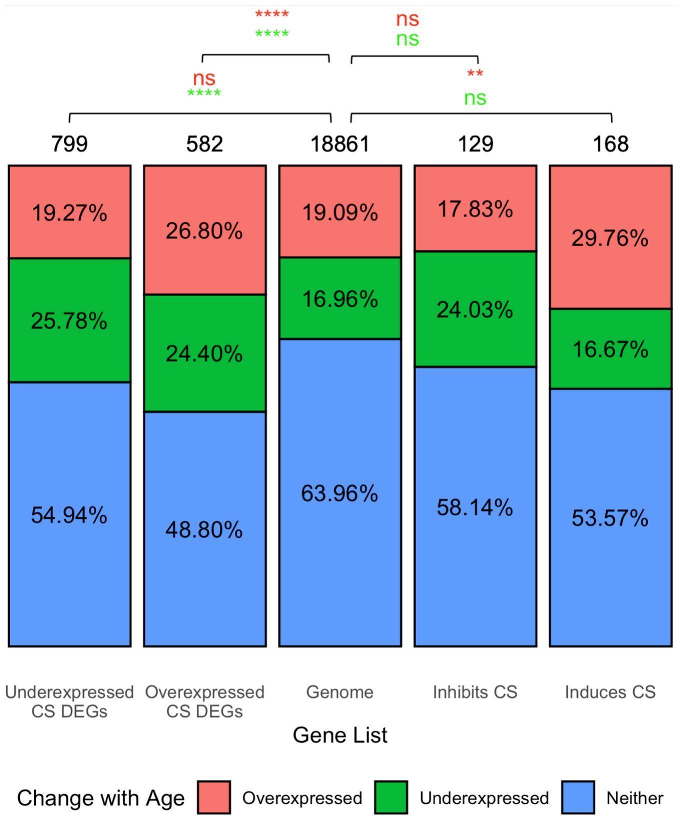

****
****
ns
ns
ns
****
**
ns
799
582
18861
129
168
19.27%
25.78%
54.94%
26.80%
24.40%
48.80%
19.09%
16.96%
63.96%
17.83%
24.03%
58.14%
29.76%
16.67%
53.57%
Underexpressed CS DEGs
Overexpressed CS DEGs
Genome
Inhibits CS
Induces CS
Gene List
Change with Age
Overexpressed
Underexpressed
Neither

Fig. S5

**C**

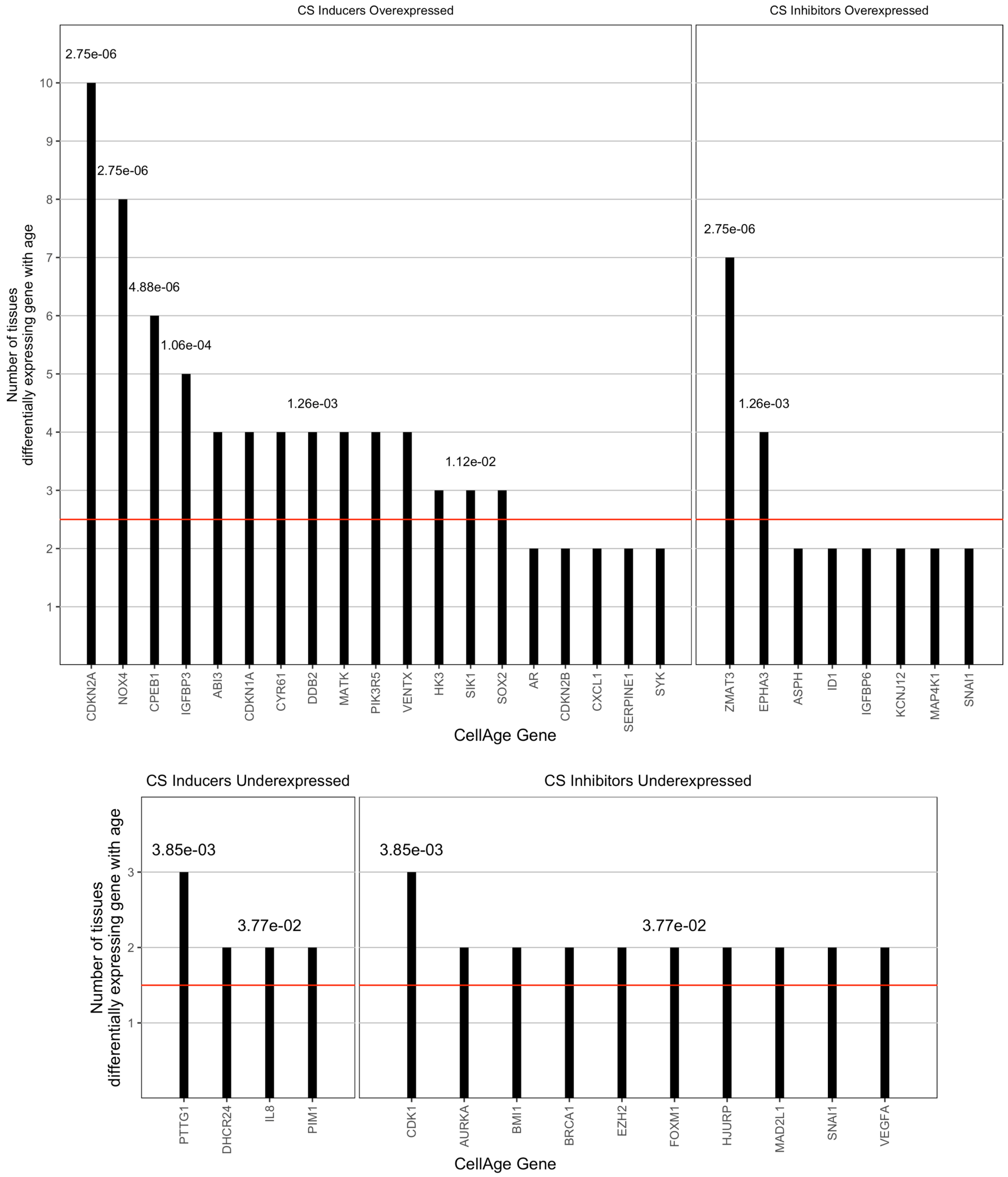

CS Inducers Overexpressed
CS Inhibitors Overexpressed
Number of tissues differentially expressing gene with age
2.75e-06
2.75e-06
4.88e-06
1.06e-04
1.26e-03
1.12e-02
2.75e-06
1.26e-03
CDKN2A
NOX4
CPEB1
IGFBP3
ABI3
CDKN1A
CYR61
DDB2
MATK
PIK3R5
VENTX
HK3
SIK1
SOX2
AR
CDKN2B
CXCL1
SERPINE1
SYK
ZMAT3
EPHA3
ASPH
ID1
IGFBP6
KCNJ12
MAP4K1
SNAI1
CellAge Gene
CS Inducers Underexpressed
CS Inhibitors Underexpressed
Number of tissues differentially expressing gene with age
3.85e-03
3.77e-02
3.85e-03
3.77e-02
PTTG1
DHCR24
IL8
PIM1
CDK1
AURKA
BMI1
BRCA1
EZH2
FOXM1
HJURP
MAD2L1
SNAI1
VEGFA
CellAge Gene


**D**

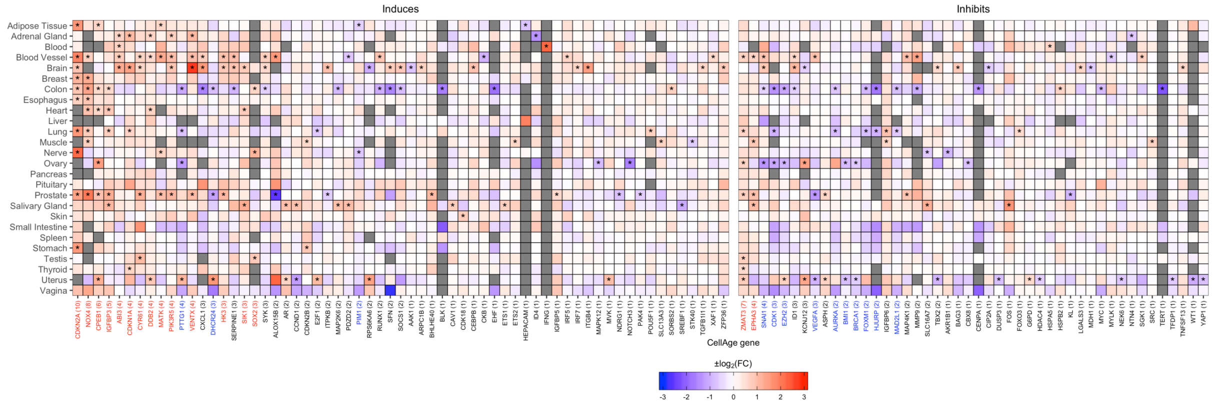

Induces
Inhibits
Adipose Tissue
Adrenal Gland
Blood
Blood Vessel
Brain
Breast
Colon
Esophagus
Heart
Liver
Lung
Muscle
Nerve
Ovary
Pancreas
Pituitary
Prostate
Salivary Gland
Skin
Small Intestine
Spleen
Stomach
Testis
Thyroid
Uterus
Vagina
CellAge gene
±log2(FC)
-3
-2
-1
0
1
2
3

Fig. S5

**Fig. S5. (A) Breakdown of CS genes differentially expressed in at least one tissue with age. Numbers over each bar represent total number of genes in each dataset. Some genes were differentially expressed in opposite directions with age across multiple tissues, and as such are listed as 'both.' (B) Comparison between ratio of CS genes differentially expressed in at least one tissue with age vs. all protein-coding genes differentially expressed in at least one tissue with age. Red values indicate comparison between overexpressed genes, whereas green values indicate comparisons between underexpressed genes. Significance assessed using Fisher's exact test with BH correction (NS – not significant, * – p<0.05, ** – p<0.01, *** – p<0.001, **** – p<0.0001). Total number of genes differ in A and B because 'both' was added to both overexpressed and underexpressed and to gene totals to compensate.** ***(C) CellAge genes differentially expressed in multiple tissues with age. Numbers indicate p-values while black bars above the red line indicate the CellAge gene was differentially expressed with age in more tissues than expected by chance (p<0.05, gene expression tissue overlap simulations with BH correction) (SI Table 16 – 19). (D) Closer look at the differential gene expression with age of CS genes in different tissues with age. Asterisks denote significant DEGs with age.***

Fig. S6

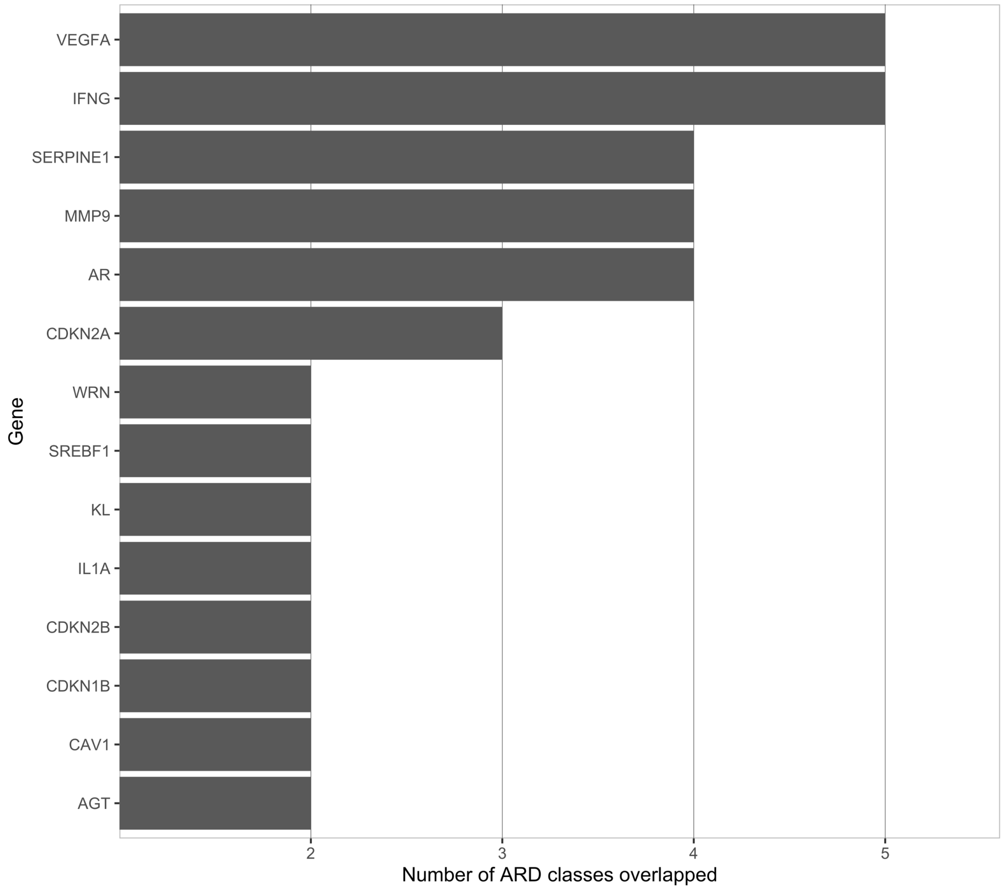


**Fig. S6. Results of CellAge genes that overlap with multiple ARD classes.**

Fig. S7

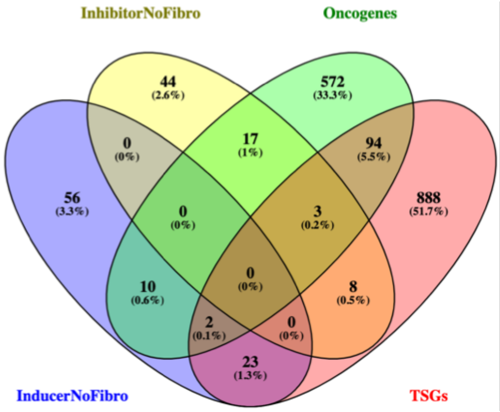


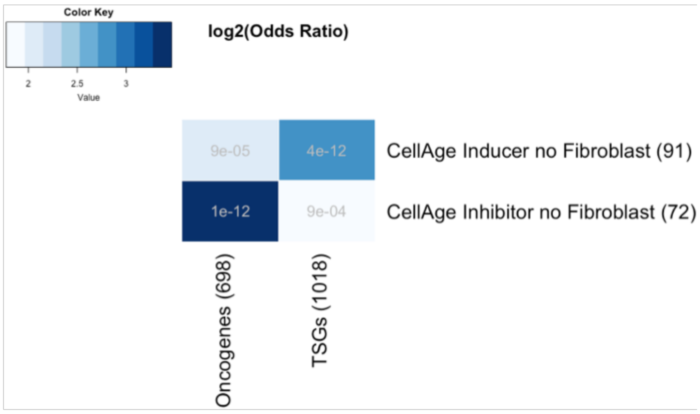


**Fig. S7. (A) Overlap between CellAge inducers and inhibitors, and oncogenes and tumour-suppressing genes after removing genes only tested in fibroblasts. (B) Adjusted p-value and odds ratio of the overlap analysis. The number of overlapping genes in each category was significant ($p<0.05$, Fisher's exact test with BH correction). P-values are shown in grey writing for each comparison.**

Fig. S8

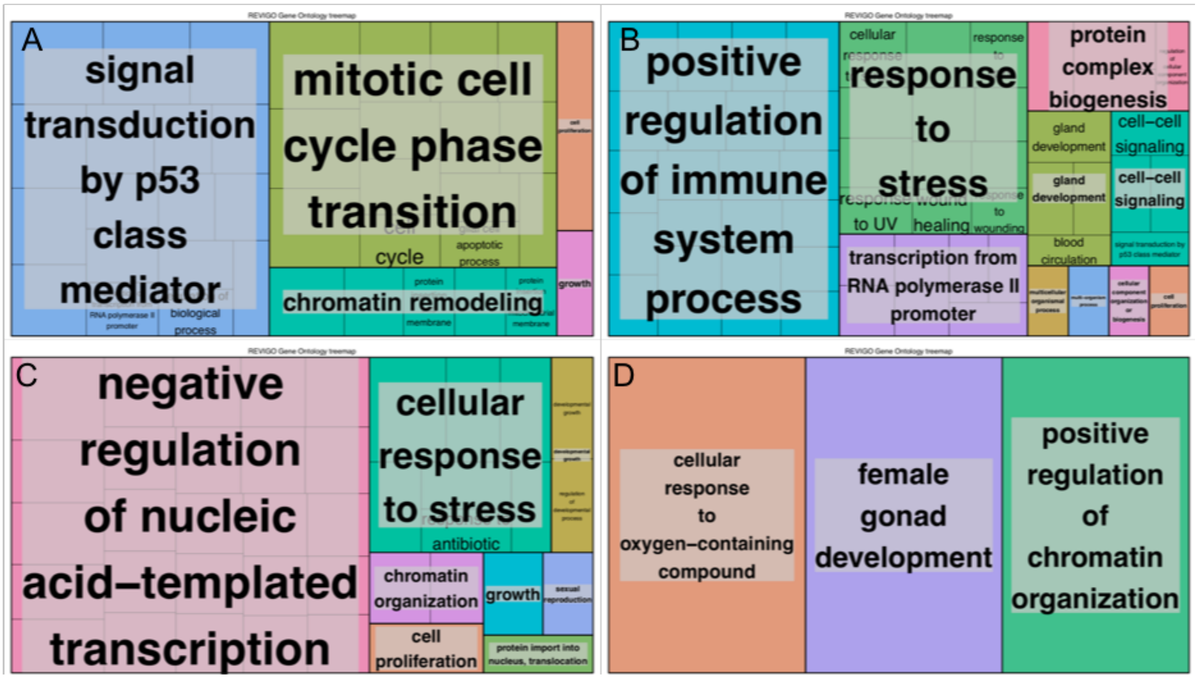


**Fig. S8. Functional enrichment of overlapping CellAge and cancer-related genes. (A) Between CellAge senescence inducer genes and TSGs, (B) Between CellAge senescence inducer genes and oncogenes. (C) Between CellAge senescence inhibitor genes and TSGs. (D) Between CellAge inhibitor genes and oncogenes.**

Fig. S9

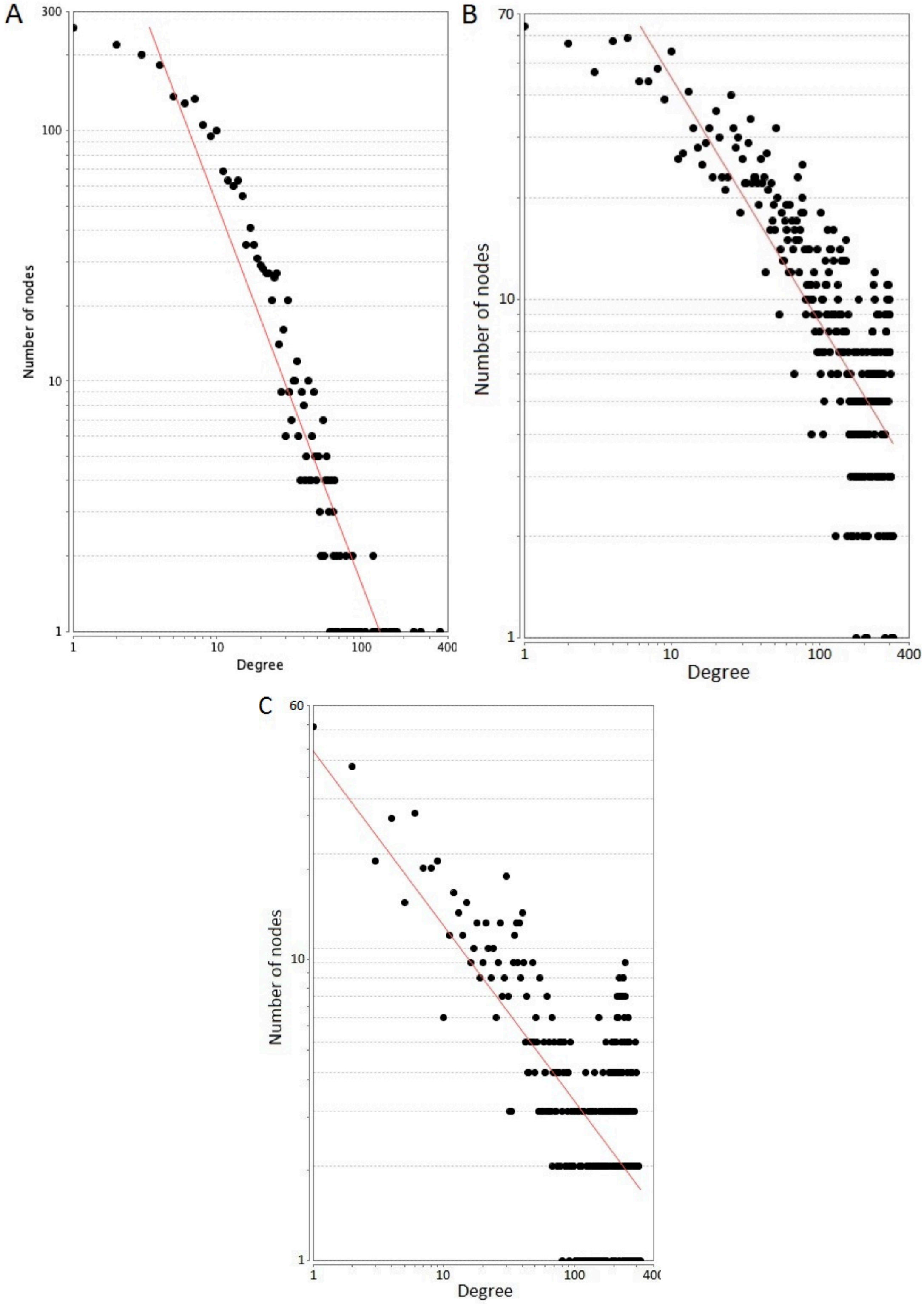


**Fig. S9. Node degree fit to the power law distribution. The fit of the node-degree distribution to a power law (red line) confirms the scale free structure of the networks. (A) The Protein-Protein Interaction network had a correlation of 0.74 and an R-squared value of 0.885. (B) The RNA-Seq**

Fig. S9

**Unweighted Co-expression Network had a correlation of 0.783 and an R-squared value of 0.630.**

**(C) The Microarray Unweighted Co-expression Network had a correlation of 0.900 and an R-squared of 0.456.**

Fig. S10

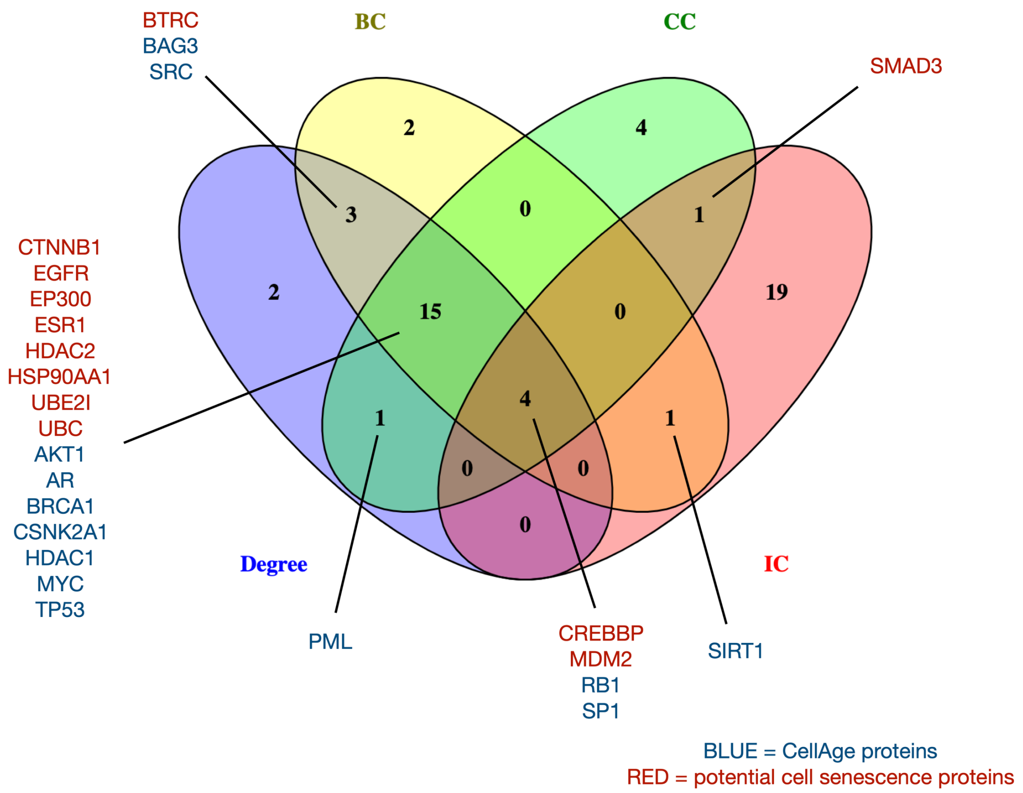


**Fig. S10. Intersection of genes occupying multiple top positions across the network topology parameters for the PPI network.**

Fig. S11

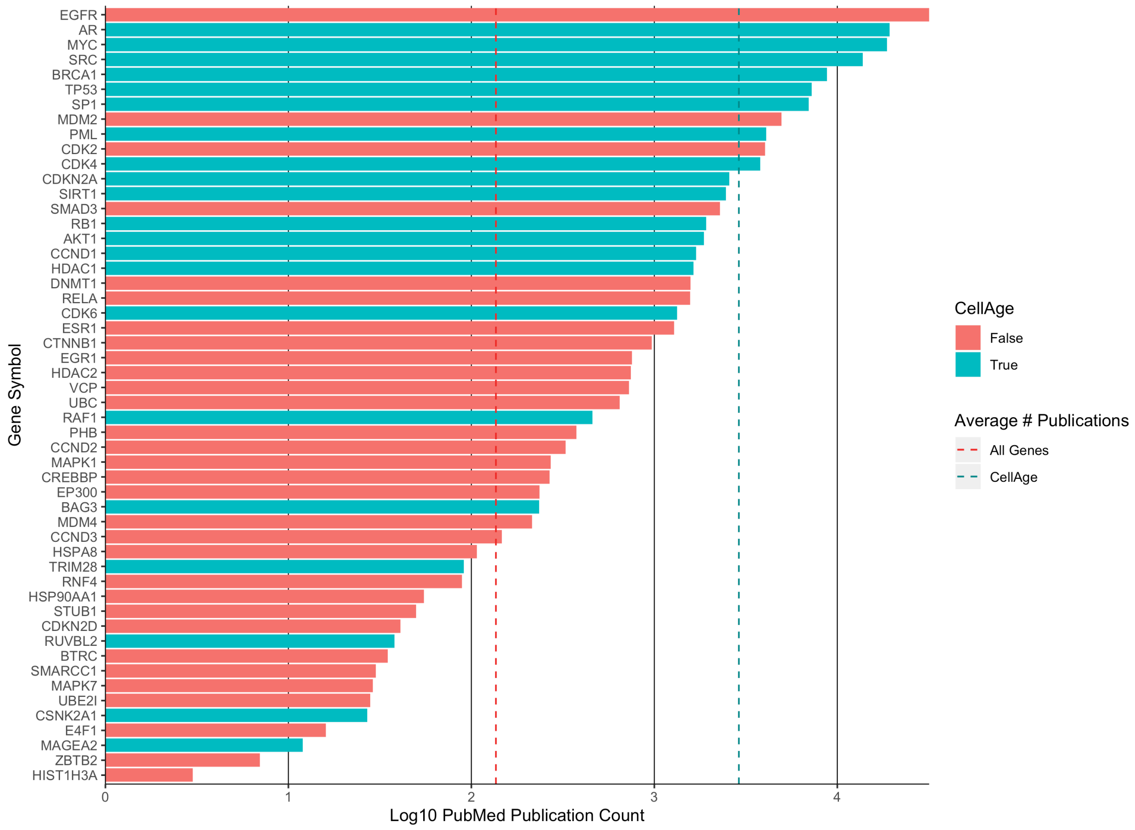


**Fig. S11. Publication counts for high centrality genes in the PPI network. The CellAge genes (blue bars), generally appear in a greater number of publications. The dashed red line shows the mean number of publications for all genes, while the dashed blue line displays the mean number of publications for CellAge genes.**

Fig. S12

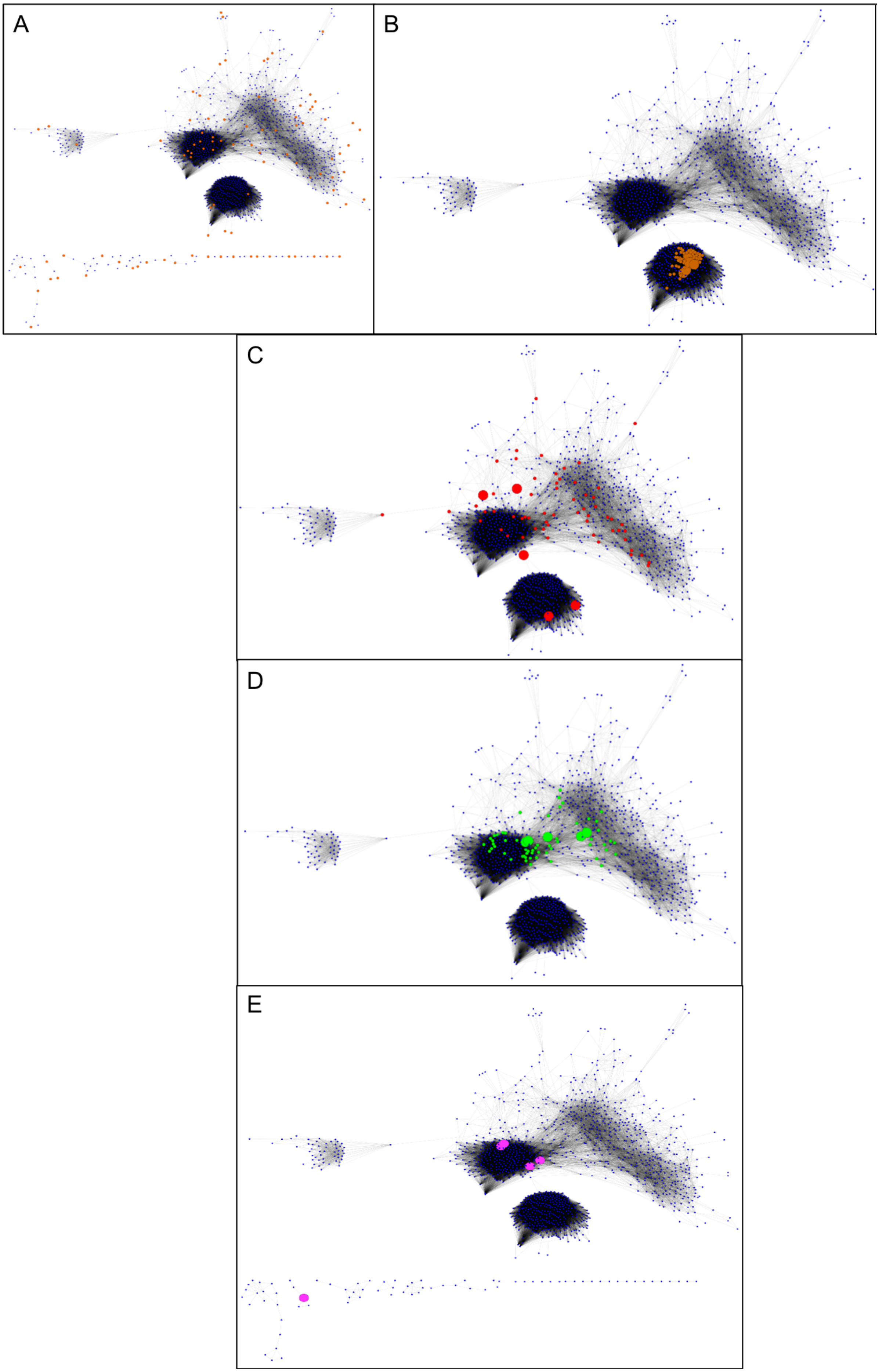

Fig. S12

**Fig. S12. The microarray co-expression network. (A) Seed Nodes of the Microarray Unweighted Co-expression Network. The 95 seed nodes, obtained from CellAge database of senescence related genes are represented in orange, size 2X. The first interacting partners are represented in blue, size 1X. The network is divided into a main component with 1198 nodes and 18 smaller components (below) with 76 nodes in total. (B) Top 5% Degree Microarray Unweighted Co-expression Network. The top 5% nodes with the largest degree of the network are represented in brown, size 2X. The top 5 nodes with the largest degree (RPRML, C11orf95, NKX2-4, UTF1 and PTH2) are highlighted in brown, size 6X. The remaining nodes are shown in blue, size 1X. All the top 5% nodes were located in the main component of the network. (C) Top 5% Betweenness Centrality (BC) Microarray Unweighted Co-expression Network. The top 5% nodes with the largest BC of the network are represented in red, size 2X. The top 5 nodes with the largest BC (DDX54, TRIM28, SPEF1, CD164L2 and SSRP1) are highlighted in red, size 6X. The remaining nodes are shown in blue, size 1X. (D) Top 5% Closeness Centrality (CC) Microarray Unweighted Co-expression Network. The top 5% nodes with the largest CC of the network are represented in green, size 2X. The top 5 nodes with the largest CC (RRM1, SUPT16H, NUP205, MCM6 and DHX9) are highlighted in green, size 6X. The remaining nodes are shown in blue, size 1X. SMC4 was also at the top 5% CC. (E) Significantly Increased Connectivity (IC) nodes in the Microarray Unweighted Co-expression Network. The 5 nodes with significant IC with genes related to cell senescence according to CellAge database are represented in pink, size 6X. The nodes with significant IC were: HMGB2, EHD2, SMC4, LMNB1 and CKAP2. The remaining nodes are shown in blue, size 1X.**

Fig. S13

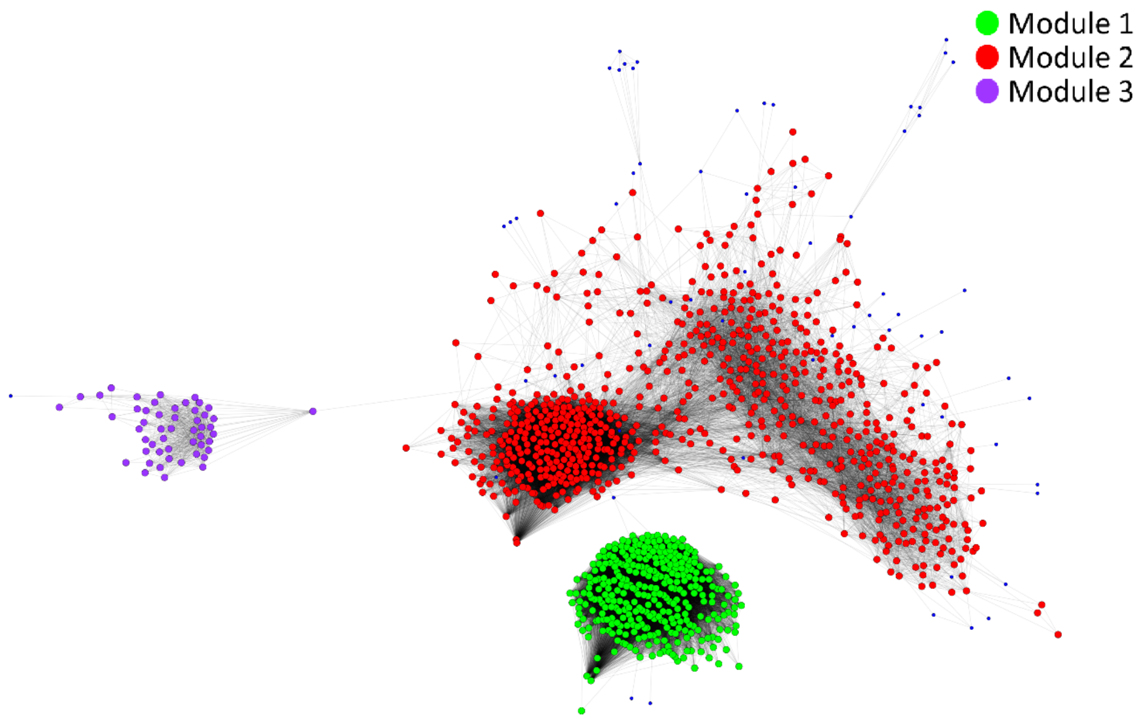


**Fig. S13. Modular analysis of the Microarray Unweighted Co-expression Network. On the top right, the legend links the nodes of each module to their assigned colours. There are three modules in the network. The modules are numbered in order of modularity.**

Fig. S14

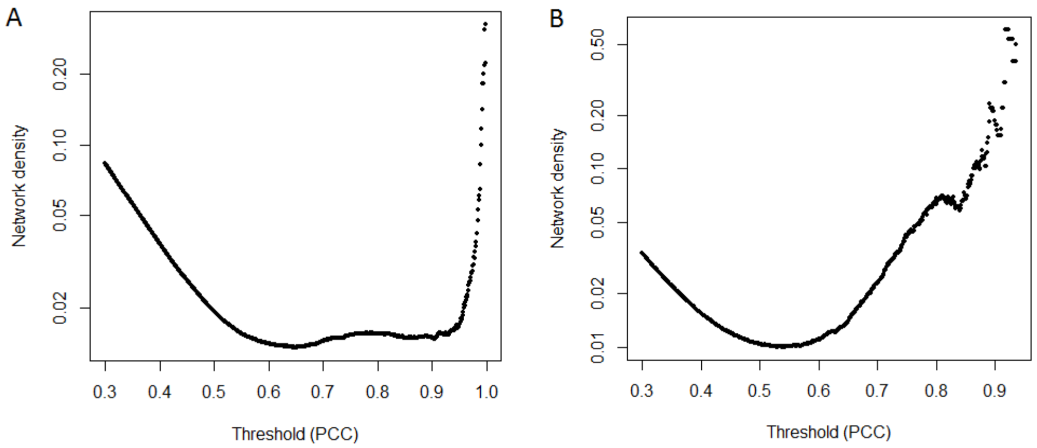


**Fig. S14. Selection of Pearson Coefficient Correlation (PCC) threshold by network density. The figures represent the network density of the database of interactions generated by applying each PCC threshold to (A) the GeneFriends Database of RNAseq co-expression correlation and (B) the COXPRESdb Database of Microarray co-expression correlation. The minimums of network densities are placed at 0.65 and 0.53 respectively.**
