# Supplementary figures and images for: A multidimensional systems biology analysis of cellular senescence in aging and disease
Source: Genome Biol. 2020 Apr 7;21:91. doi: 10.1186/s13059-020-01990-9 (PMC7333371; doi:10.1186/s13059-020-01990-9)

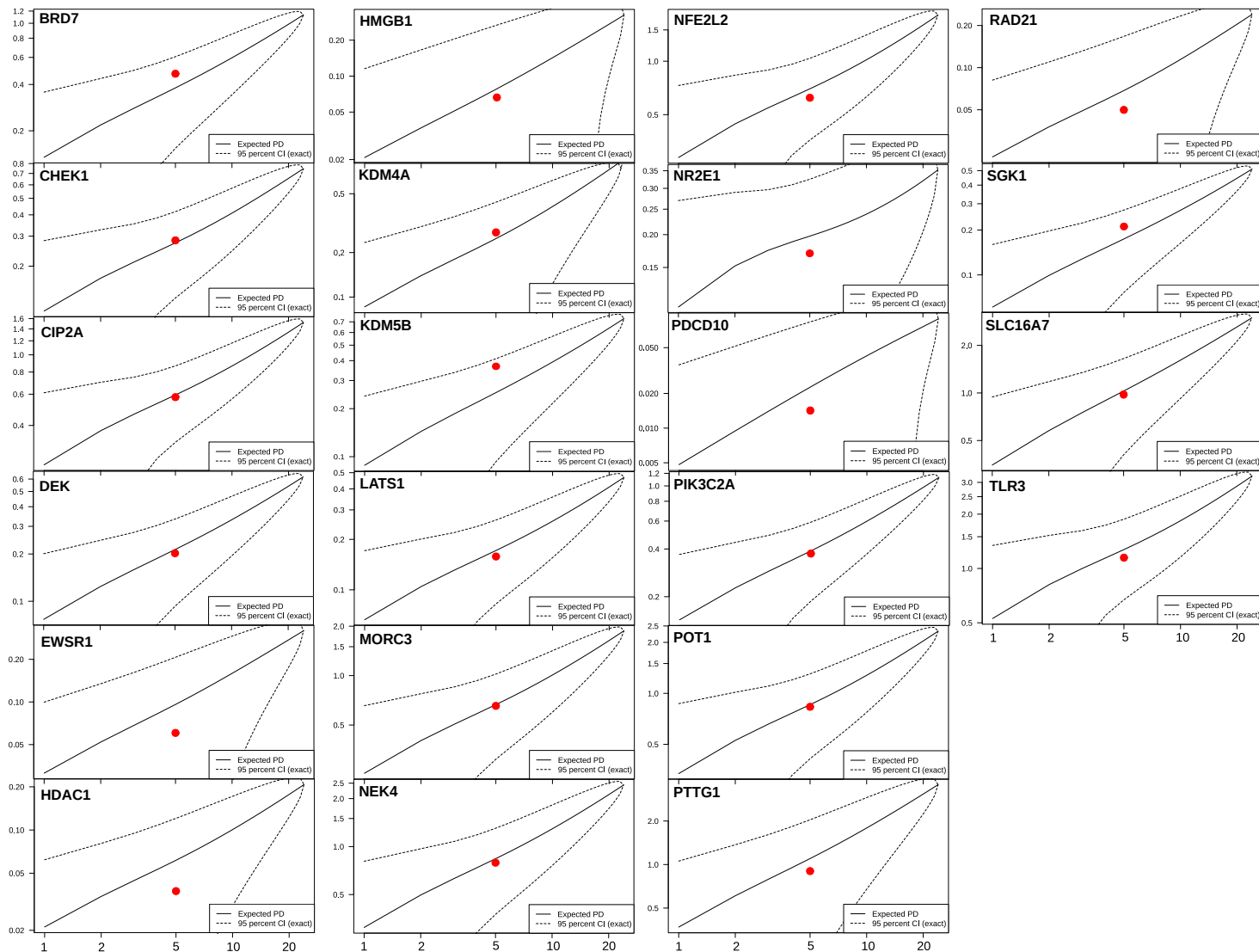

Supplement: Supplementary file 4 — Evolutionary distance in CellAge genes. PDF with Faith’s phylogenetic diversity index of 22 individual CellAge genes conserved amongst all 24 mammalian species. [file 13059_2020_1990_MOESM4_ESM.pdf]
